# Seasonal Adaptation: Geographic Photoperiod–Temperature Patterns Explain Genetic Variation in the Common Vole Tsh Receptor

**DOI:** 10.3390/genes14020292

**Published:** 2023-01-22

**Authors:** Laura van Rosmalen, Robin Schepers, Wensi Hao, Anna S. Przybylska-Piech, Jeremy S. Herman, Joanna Stojak, Jan M. Wójcik, Louis van de Zande, Jeremy B. Searle, Roelof A. Hut

**Affiliations:** 1 Chronobiology Unit, Groningen Institute for Evolutionary Life Sciences, University of Groningen, 9747 Groningen, The Netherlands; 2 Department of Natural Sciences, National Museums Scotland, Edinburgh EH1 1JF, UK; 3 Mammal Research Institute, Polish Academy of Sciences, 17-230 Białowieża, Poland; 4 Department of Experimental Embryology, Institute of Genetics and Animal Biotechnology, Polish Academy of Sciences, Jastrzębiec, 05-552 Magdalenka, Poland; 5 Evolutionary Genetics, Development and Behaviour, Groningen Institute of Evolutionary Life Sciences, University of Groningen, 9747 Groningen, The Netherlands; 6 Department of Ecology and Evolutionary Biology, Cornell University, Ithaca, NY 14853, USA

**Keywords:** seasonal reproduction, Tsh receptor, temperature–photoperiod ellipsoid, natural selection, *Microtus arvalis*, common vole, climate change

## Abstract

The vertebrate photoperiodic neuroendocrine system uses the photoperiod as a proxy to time the annual rhythms in reproduction. The thyrotropin receptor (TSHR) is a key protein in the mammalian seasonal reproduction pathway. Its abundance and function can tune sensitivity to the photoperiod. To investigate seasonal adaptation in mammals, the hinge region and the first part of the transmembrane domain of the *Tshr* gene were sequenced for 278 common vole (*Microtus arvalis*) specimens from 15 localities in Western Europe and 28 localities in Eastern Europe. Forty-nine single nucleotide polymorphisms (SNPs; twenty-two intronic and twenty-seven exonic) were found, with a weak or lack of correlation with pairwise geographical distance, latitude, longitude, and altitude. By applying a temperature threshold to the local photoperiod–temperature ellipsoid, we obtained a predicted critical photoperiod (pCPP) as a proxy for the spring onset of local primary food production (grass). The obtained pCPP explains the distribution of the genetic variation in *Tshr* in Western Europe through highly significant correlations with five intronic and seven exonic SNPs. The relationship between pCPP and SNPs was lacking in Eastern Europe. Thus, *Tshr*, which plays a pivotal role in the sensitivity of the mammalian photoperiodic neuroendocrine system, was targeted by natural selection in Western European vole populations, resulting in the optimized timing of seasonal reproduction.

## 1. Introduction

Herbivores in mid to high latitudes are dependent on seasonal food availability and use the photoperiod (i.e., day length) to synchronize their reproduction with primary food production [1]. Primary production in the food web is temperature-dependent [2,3,4]. Since temperature is a notoriously noisy environmental signal, herbivores use the photoperiod as a proxy to adjust the seasonal timing of reproduction [1]. The annual relationship between the photoperiod and ambient temperature is primarily dependent on latitude [5] but also changes with altitude and longitude, depending on local climatic conditions. As a consequence, for the Northern Hemisphere, primary food production and the biological spring generally start later in the year at higher latitudes, coinciding with the longer photoperiods than at low latitudes. Therefore, selection pressure on the timing of reproduction has caused the adaptive evolution of the seasonal timing mechanisms in herbivores, such that reproduction in spring starts with the longer photoperiods in the more northern populations in the Northern Hemisphere.

Latitudinal clines in annual timing were described in many insect species and in some bird and plant species but rarely in mammals [5]. For the photoperiodic induction of diapause in insects, the critical photoperiod (CPP) increases with latitude [5,6,7,8,9,10]. Furthermore, bird species change their annual breeding frequency patterns at different latitudes, with the peak shifting to a later time point in the year at higher latitudes [1]. These findings suggest that there is a latitudinal adaptation of the photoperiodic timing mechanisms driving reproduction. A genetic basis for variation in photoperiodic responsiveness was demonstrated in the parasitoid *Nasonia vitripennis*, where it is associated with the clinal allelic variation of the *period* gene [11,12], in pitcher-plant mosquitoes *Wyeomyia smithii* [13,14,15], in deer mice *Peromyscus leucopus* [16] and *Peromyscus maniculatus* [17], and in Siberian hamsters *Phodopus sungorus* [18,19]. Although the latitude of origin influences the photoperiodic responses in deer mice [20], the underlying genetics for the adaptation of such photoperiodic mechanisms in mammals is not clear.

Laboratory experiments revealed that annual rhythms in physiology and reproduction are driven by the photoperiodic neuroendocrine system [21]. This mechanism is well-conserved among vertebrates, including the common vole, *Microtus arvalis* (Pallas 1778) [22,23,24,25]. The distribution of *Microtus* ranges from close to the equator to arctic regions (14° N–72° N) in North America, Europe, and northern Asia, which makes it an excellent genus to study the latitudinal adaptation of the photoperiodic system. Perhaps the primary logical reason why small terrestrial mammals should be ideal for using latitudinal clines as a method to study the evolution of photoperiodic adaptation is because they do not show (large-scale) migration like marine mammals, birds, and some insects do.

The photoperiodic neuroendocrine system comprises a seasonal timing mechanism that synchronizes to changes in the photoperiod using the central circadian clock (i.e., the suprachiasmatic nucleus; SCN). The photoperiod is inversely related to the duration of the nocturnal melatonin release by the pineal gland [26,27,28]. Under a short photoperiod, pineal melatonin is present in the morning hours (12h after lights off) and binds to melatonin receptors in the pars tuberalis of the pituitary, causing suppression of the thyroid-stimulating hormone β-subunit (TSHβ) [29,30]. Under a long photoperiod, melatonin is absent in the morning (12h after lights off), which allows the transcriptional coactivator Eyes absent 3 (EYA3) and, subsequently, TSHβ to increase. The TSHβ forms an active heterodimer with the glycoprotein hormone α-subunit (αGSU) [31], which then binds as TSH to its receptor, the TSHR, in the tanycytes, a key population of cells involved in the neuroendocrine control of reproduction, around the third ventricle of the brain, where it increases the production of iodothyronine deiodinase 2 (DIO2) [32,33,34,35,36]. This leads to increased active thyroid hormone levels (T_3_) [37], which indirectly permit gonadotropin-releasing hormone (GnRH) release into the hypophysial portal system to control reproductive behavior [33,34,35].

TSH-dependent sensitivity to the photoperiod, which is defined as a shift of the photoperiodic response curves, can be modulated by TSHR abundance and function. The TSHR is, therefore, an essential protein in the mammalian seasonal reproduction pathway. In addition, Ho and colleagues showed that *Tshr* mutations can change the signaling efficiency of the receptor [38]. Selection on the *Tshr* gene, and closely located regions, has been shown in the domestic chicken (*Gallus gallus domesticus*) [39] and sheep (*Ovis aries*), respectively [40,41], which suggests that the transmembrane domain is especially important in modulating the photoperiodic control of reproduction [39,42]. Therefore, to assess the seasonal adaptation of the photoperiodic mechanisms, we focus on *Tshr*.

The protein encoded by the *Tshr* gene belongs to the glycoprotein hormone receptor family [43]. The TSHR is a seven-transmembrane domain (TMD) G protein-coupled receptor (GPCR) with a large extracellular N-terminal part, containing leucine-rich repeats (LRRs). LRRs form a hormone-binding pocket and are responsible for TSH recognition and binding [44]. TSH binding causes a conformational change in the TSHR, which activates G-protein-dependent signaling transduction. The extracellular domain containing LRRs is connected to a large transmembrane helix, the hinge region.

The *Tshr* gene is very large, owing to its large introns. This offers many possibilities for cis-regulatory elements that may modulate transcription [45]. Mutations in intronic regions may disrupt transcription factor binding, which may lead to altered *Tshr* expression. Human *TSHR* mRNA splice variants encoding a TSHR without TMD have been reported [46], which may hint at alternative splice sites within intronic regions prior to the exon encoding for the TMD. Since the hinge region and the first TMD are important for ligand binding and for signaling transduction [47], and mutations in the hinge region of the *TSHR* are known to change the signaling efficiency of the receptor [38], we consider this region as a potential target for natural selection and functional adaptive variation.

**Figure 1:**
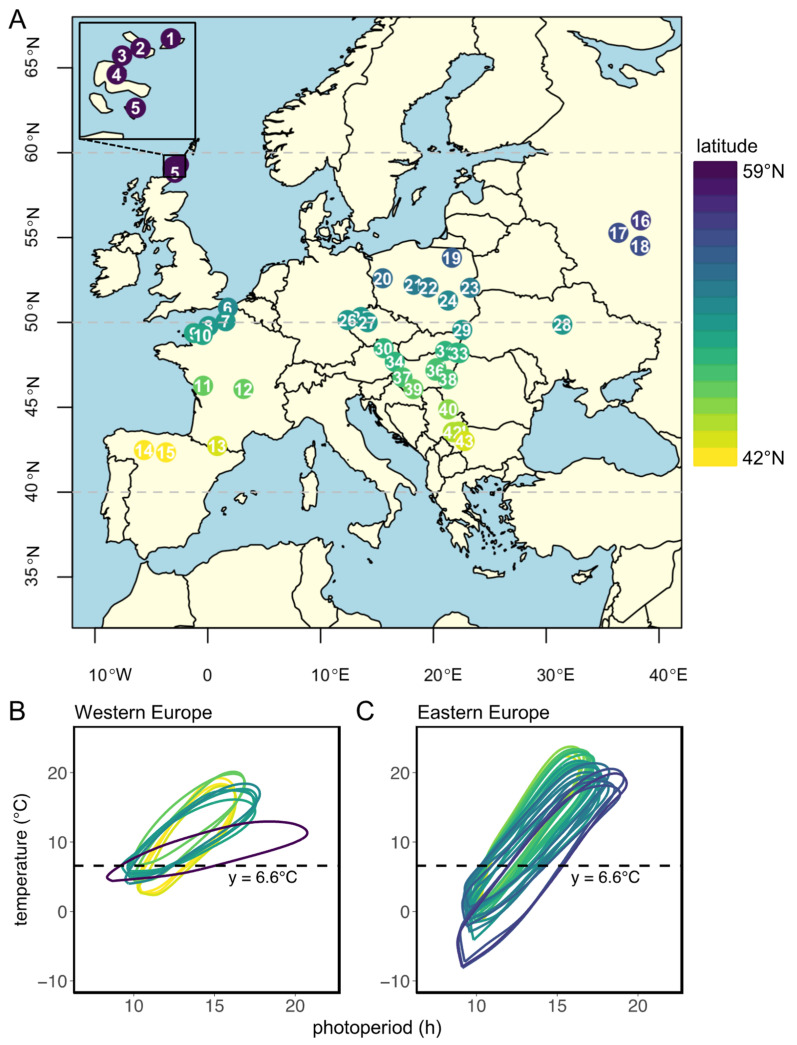
Sample locations and local annual photoperiod–temperature ellipsoids. (**A**) Distribution map of localities of common vole samples used in this study. Location numbers with corresponding environmental proxies are listed in Table S1. (**B**) Annual patterns (counter-clockwise) of photoperiod and 10-year average monthly ambient temperature for each sample location in Western Europe and (**C**) in Eastern Europe. Temperature data were obtained from the closest weather station (always within 110 km of sample location) obtained from Wunderground (https://www.wunderground.com/ (accessed on 7 December 2022)). Photoperiod was obtained from https://www.timeanddate.com (accessed on 7 December 2022) and is based on civil twilight times at dawn and dusk, which is the timing at which log light intensities change most rapidly [5,48]. Civil twilight incorporates geographical and seasonal variation in the duration of twilight [49] and is, therefore, considered as the moment of ‘lights on’ and ‘lights off’ for biological systems [5]. Dotted lines indicate a temperature threshold at 6.6 °C, from which the corresponding predicted critical photoperiod (pCPP) in spring (ellipse crossing the 6.6 °C line for the second time) is used as a proxy for onset of grass growth and, consequently, as a proxy for onset of the favorable season. Regional pCPPs are listed in Table S1. Colors indicate latitude, ranging from 42° N (yellow) to 59° N (purple).

In this study, we evaluate the genetic adaptation to local climate conditions in the common vole *Tshr* gene by comparing genetic variance over the large European geographical range of this herbivorous rodent. For this purpose, we focus on the end of intron 8 and the beginning of exon 9, encoding for the hinge region and the first part of the transmembrane domain of the TSHR. Since the reproductive response at high latitudes in the Northern Hemisphere requires longer photoperiods, and, therefore, higher TSH levels, the allelic variation of the *Tshr* gene may be associated with reduced TSHR signaling at high latitudes. Tissue samples were collected from 43 different localities over a large European geographical distribution (Figure 1A, Table S1). The large variations in latitude (42°21′36″ N–59°17′60″ N), longitude (5°31′48″ W–38°23′24″ E), and altitude (4–2146 m above sea level) allowed us to assess whether location-specific annual photoperiod–temperature ellipsoid patterns can explain the distribution of the genetic variation in the coding and non-coding parts of *Tshr*.

## 2. Materials and Methods

### 2.1. Tissue Samples

We obtained tissue samples from 278 previously collected specimens of female and male common voles from both Western Europe (France, Great Britain, and Spain) and Eastern Europe (Czech Republic, Hungary, Poland, Russia, Serbia, and Ukraine) [50,51] (Figure 1A, Table S1). Specimens were collected between 1995 and 2015 (voles from the same site were caught simultaneously) and consisted of appendages (legs, toes, ears, tail tips, and muscle tissues) preserved in 96–99% ethanol at 4–15 °C. Sex was not documented for all specimens; therefore, we could not account for sex in the model.

### 2.2. DNA Isolation, PCR and Sanger Sequencing

Total genomic DNA was isolated using the DNeasy Blood and Tissue Kit (Qiagen, Hilden, Germany) for Western European samples and using the Syngen Tissue DNA Mini Kit (Syngen Biotech, Wrocław, Poland) for Eastern European samples, in accordance with the protocols of the manufacturer. Primers (Table S2) were designed based on the common vole genome (NCBI:txid47230, GCA_007455615.1) using Primer-BLAST (NCBI), in two overlapping fragments, each 1100-1200 bp in length. Using those primers, the last ~829 bp of intron 8 and the first ~849 bp of exon 9 of the *Tshr* gene were amplified by PCR using DreamTaq (Thermoscientific^TM^, Waltham, MA, USA). A mastermix containing 15.7 μL ultrapure H_2_O, 2 μL Dreamtaq Buffer (10×), 0.4 μL dNTP mix (10 mM), 0.4 μL Forward primer (10 μM), 0.4 μL Reverse primer (10 μM), and 0.1 μL DreamTaq DNA polymerase (5 U/μL) was prepared for each reaction. Twenty μL-reactions (1 μL DNA + 19 μL mastermix) were carried out for each sample by using a thermalcycler (S1000^TM^, Bio-Rad, Hercules, CA, USA) (Table S3). Following PCR, an enzymatic clean-up with ExoSAP-IT reagent (Applied Biosystems^TM^, Foster City, CA, USA) was performed in order to remove excess primers and nucleotides. Then, 5 μL of cleaned PCR product and 5 μL of the forward or reverse primer (5 μM) were transferred to a new 1.5 mL tube and sent out for Sanger sequencing (Eurofins Genomics, Ebersberg, Germany). The intronic fragment was sequenced in two directions, while the exonic fragment was sequenced only in the forward direction.

### 2.3. Data Analysis and Statistical Analysis

The distribution map of common vole samples used in this study (Figure 1A) was generated using the following R packages: ‘rworldmap’ [52], ‘rworldxtra’ [53], ‘RcolorBrewer’ [54], ‘maptools’ [55], and ‘classInt’ [56]. Ellipse-like annual relationships between temperature and photoperiod (Figure 1B,C) were built using ~10-year (between 2000 and 2019) average monthly ambient temperatures obtained from local weather stations (within 110 km of sample location) at http://www.wunderground.com (accessed on 7 December 2022). Photoperiods, based on civil twilight times at dawn and dusk at different locations, were retrieved from https://www.timeanddate.com/ (accessed on 7 December 2022). Grass growth in spring is used as a proxy for the onset of the favorable reproductive season. Grass growth is initiated at 5–10 °C air temperature [3,57,58]. To include all locations in our analysis, a temperature threshold at 6.6 °C was used to deduce, for further analysis, the corresponding predicted critical photoperiod (pCPP) that would initiate optimal timing of reproduction.

**Figure 2:**
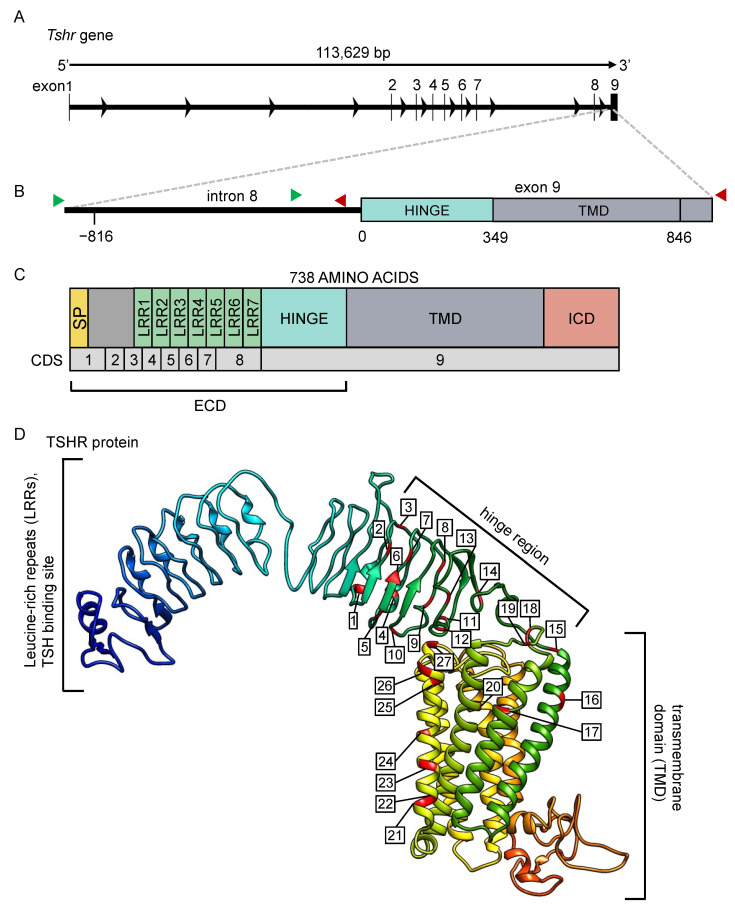
The *Tshr* gene and predicted protein for the common vole. (**A**) The *Tshr* gene for the common vole, consisting of 8 introns and 9 exons. (**B**) The magnified region, including the last part of intron 8 and the first part of exon 9, were sequenced in this study. Green arrows indicate location of forward primers, and red arrows indicate location of reverse primers for sequencing. (**C**) The predicted TSHR protein and (**D**) its 3D structure. All mutations found in the current study are labeled and listed in Tables S4–S7. SP = signal peptide, LRR = leucine-rich repeat, TMD = transmembrane domain, ICD = intracellular domain, ECD = extracellular domain, CDS = coding sequence.

The ‘Phyre2’ web portal for protein modeling was used to predict the TSHR protein 3D structure (Figure 2D) [59]. SNPs were detected by sequence alignments using ‘CLC Sequence Viewer’ (version 8.0) (QIAGEN, Aarhus, Denmark). Chromatograms were checked for sequencing quality and heterozygosity of SNPs in the Mac OS software ‘4-peaks’ (Nucleobytes, Aalsmeer, The Netherlands). Variation in DNA sequences were classified as SNPs if >3 of the specimens contained the mutation. Putative transcription factor bindings sites were predicted using AliBaba2 [60]. To statistically test gene–environment associations, we used a population-based approach, in which an environmental variable was modeled as a linear function of population allele frequency [61]. Pearson’s correlation tests were carried out: pairwise distances of allele frequencies correlated with pairwise geographical distance, pairwise latitudinal difference, pairwise longitudinal difference, pairwise altitudinal difference, and pairwise critical photoperiod difference. *p*-values were adjusted according to the Benjamini–Hochberg procedure [62,63], which is one of the strongly recommended methods to use in environmental association analysis [61]. Pairwise linkage disequilibrium heatmaps (Figure S3) were generated using the R-package ‘LDheatmap’ [64]. The constructed phylogenetic tree (Figure S2) from SNP frequency data by using the neighbor-joining method [65] was generated using ‘POPTREEW’ [66]. All other analyses were performed using ‘RStudio’ (version 1.2.1335), and figures were generated using the R-package ‘ggplot2’ [67].

## 3. Results

The *Tshr* gene of the common vole is 113,629 bp long and consists of eight introns and nine exons (Figure 2A). In this study, we sequenced a ~1700 bp region around the beginning of exon 9, comprising 829 bp of intronic and 849 bp of exonic sequences (Figure 2B). The predicted TSHR protein structure, based on the *M. arvalis* genome (NCBI: txid47230, GCA_007455615.1), comprises seven leucine-rich repeats, a hinge region, and seven transmembrane domains (Figure 2C,D).

**Figure 3:**
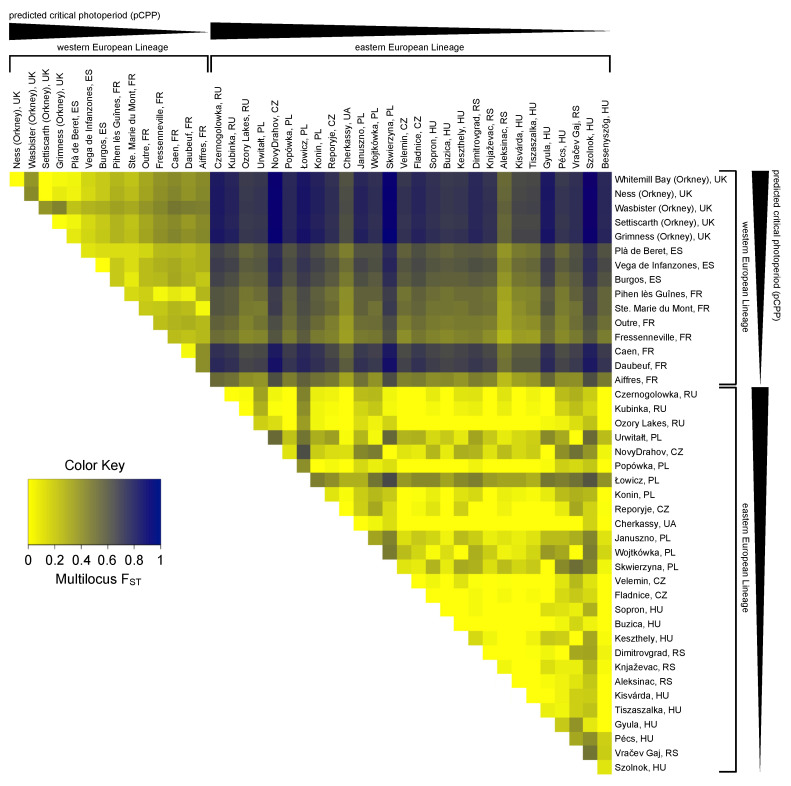
Pairwise multilocus F_ST_ heatmap for *Tshr* haplotypes. F_ST_ values were calculated using the original method for estimation of genetic distance with correction for sample size bias [70]. Colors indicate pairwise multilocus F_ST_ values ranging from 0 (yellow) to 1 (dark blue). Western and Eastern European populations are ordered from long to short predicted critical photoperiods (pCPPs).

Forty-nine single nucleotide polymorphisms (SNPs) were detected (Tables S4 and S5). Twenty-two intronic SNPs and twenty-seven exonic SNPs were found, from which twenty-three were synonymous. These SNPs were used to calculate the genetic differentiation of the sampled populations. The pairwise multilocus fixation index (F_ST_) estimates ranged from F_ST_ = 0.000 to 0.978 (mean F_ST_ = 0.366; with 0 denoting no difference and 1 referring to completely different populations) and revealed a high genetic differentiation between the sampled populations (Figure 3). The high F_ST_ values in this species are in agreement with previous studies [68,69]. The structure of the observed population differentiation suggests that not only population subdivision but also natural selection may be an explanatory factor. Therefore, it was tested whether the observed SNPs are associated with geographical location. The genetic differentiation between the Eastern and Western European populations was larger (F_ST_ = 0.247 to 0.978; mean: 0.640) than the differentiation among Western European populations (F_ST_ = 0.016 to 0.467; mean: 0.234) and among Eastern European populations (F_ST_ = 0.000 to 0.648; mean: 0.132), and strongly depended on geographical distance and longitude (Figure 3 and Figure S2, Table S5). The constructed distance tree based on *Tshr* haplotypes (Figure S2), together with pairwise multilocus F_ST_ analysis (Figure 3), confirmed that the Western and Eastern European populations belong to different genetic lineages. Given the genetic separation of these lineages, it is appropriate to analyze Western and Eastern European samples separately. To reveal possible patterns of association between SNPs, heatmaps of the pairwise linkage disequilibrium (LD) measurements were generated (Figure S3).

### 3.1. Western European Lineage

**Figure 4:**
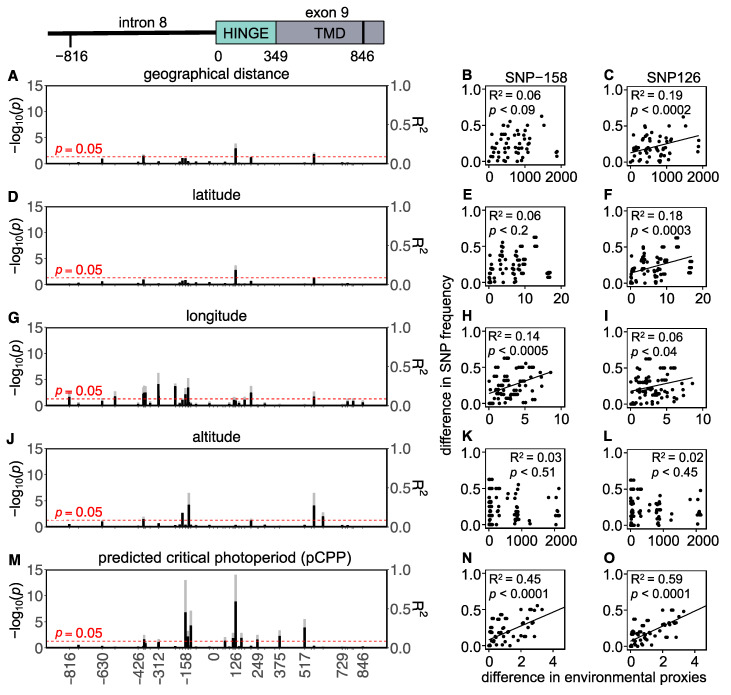
*Tshr* mutations in Western European common vole populations. Manhattan-type plots (−log_10_(*p*)) for the sequenced *Tshr* region for (**A**) geographical distance, (**D**) latitude, (**G**) longitude, (**J**) altitude, and (**M**) predicted critical photoperiod (pCPP). Gray bars indicate Benjamini–Hochberg adjusted *p*-values, and black bars indicate R^2^-values. SNPs that meet the threshold for significant correlations (*p* < 0.05) cross the red dashed line. Pairwise differences in SNP frequency for two representative mutations (SNP-158 and SNP126) related to (**B**,**C**) pairwise geographical distance, (**E**,**F**) pairwise latitudinal difference, (**H**,**I**) pairwise longitudinal difference, (**K**,**L**) pairwise altitudinal difference, and (**N**,**O**) pairwise difference in pCPP. Significant correlations are indicated by linear regression lines. All statistic results of linear models for SNP frequency related to environmental proxies are found in Table S6.

In total, 43 SNPs (22 intronic and 21 exonic) were found in the Western European populations, of which 5 were non-synonymous and 16 were synonymous (Figure 4 and Table S6). To assess whether the variation in the *Tshr* gene can be explained by local seasons, SNP frequencies correlated with different environmental proxies. The non-synonymous SNPs were not associated with the tested environmental proxies (Table S6). In the Western European lineage, 3/43 SNPs (exonic, synonymous) significantly correlated with pairwise geographical distance (Figure 4A,C, Table S6), indicating that there was a lack of regional equilibrium [71] and that an alternative approach may be used to detect selection, by classifying SNPs that show clinal variation [72]. SNP frequency weakly correlated with pairwise latitudinal, longitudinal, and altitudinal difference for the majority of the observed SNPs (latitude: 2 exonic SNPs; longitude: 11 intronic and 5 exonic SNPs; altitude: 3 intronic and 3 exonic SNP) (Figure 4D–L, Table S6). These findings show that geographical distance, latitude, and altitude by themselves are bad predictors for genetic variation in the *Tshr* gene, despite the fact that the annual photoperiod–food abundance patterns depend on all these parameters. Therefore, for each sample location, the pCPP at which grass growth is initiated in spring (at 5–10 °C ambient temperature [3,57,58]) was deduced from the local annual photoperiod–ambient temperature ellipsoids. The pCPP at 6.6 °C included ellipsoids from all sample locations. Therefore, the temperature threshold for grass-growth initiation in spring was set at 6.6 °C, and was used to deduce the corresponding pCPP, which was calculated to vary between 10.19 and 15.40 h of light/24 h (Figure 1 and Table S1). In the Western European samples, five intronic and seven exonic SNPs strongly correlated with the pairwise difference in pCPP (Figure 4M, Table S6). The F_ST_ values for these specific SNPs were high (ranging from F_ST_ = 0.032 to 0.310; mean: 0.166). All these significant mutations were, however, synonymous SNPs. The strongest associations with pCPP were found for intronic SNP-158 (G > C), −128 (T > C) and exonic SNP126 (A > G) (Figure 4M–O, Table S6). It is expected that between Orkney Island and the mainland, some of the variation reflects isolation and genetic drift. Therefore, the same analysis was performed excluding the Orkney Island populations, which revealed similar results. The pairwise multilocus F_ST_-values were high for populations that highly differed in pCPP, while the F_ST_-values were low for populations with a similar pCPP (Figure 3).

### 3.2. Eastern European Lineage

**Figure 5:**
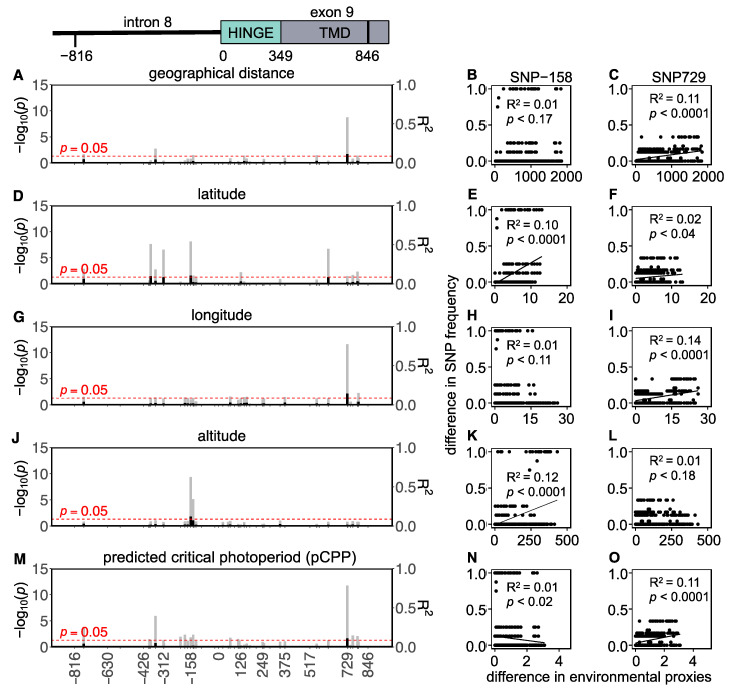
*Tshr* mutations in Eastern European common vole populations. Manhattan-type plots (−log_10_(*p*)) for the sequenced *Tshr* region for (**A**) geographical distance, (**D**) latitude, (**G**) longitude, (**J**) altitude, and (**M**) predicted critical photoperiod (pCPP). Gray bars indicate Benjamini–Hochberg adjusted *p*-values, and black bars indicate R^2^-values. SNPs that meet the threshold for significant correlations (*p* < 0.05) cross the red dashed line. Pairwise differences in SNP frequency for two representative mutations (SNP-158 and SNP729) related to (**B**,**C**) pairwise geographical distance, (**E**,**F**) pairwise latitudinal difference, (**H**,**I**) pairwise longitudinal difference, (**K**,**L**) pairwise altitudinal difference, and (**N**,**O**) pairwise difference in pCPP. Significant correlations are indicated by linear regression lines. All statistic results of linear models for SNP frequency related to environmental proxies are found in Table S7.

In total, 34 SNPs (14 intronic and 20 exonic) were found in the Eastern European populations, of which 3 were non-synonymous and 17 were synonymous (Figure 5 and Table S7). Although highly significant *p*-values for some correlations between the *Tshr* SNP frequencies and environmental proxies were found in the Eastern European lineage, the R^2^-values were extremely low (0.1 ± 0.02; mean ± SD). This indicates that in Eastern European voles, the *Tshr* SNP frequencies weakly correlate with geographical distance, latitude, longitude, altitude, and pCPP (Figure 5, Table S7). This observation indicates that genetic *Tshr* variation in Eastern Europe is unlikely to be explained by natural selection due to seasonal variance.

## 4. Discussion

Common vole populations are characterized by large-scale genetic differentiation of *Tshr*, reflecting local adaptation to annual temperature–photoperiod patterns, rather than latitude per se. Variation in *Tshr* sequence indicates that the *M. arvalis* population can be subdivided into Eastern and Western European clusters, indicating that they may belong to distinct genetic lineages (Figure 3 and Figure S3). This phylogeographical structure is consistent with those found for mitochondrial cytochrome *b* gene sequences and microsatellite loci (representing nuclear DNA) [50,51,68,73,74,75,76], which justifies analyzing the Western and Eastern European populations separately. The Western versus Eastern European divide could well be due to the reinvasion of Northern Europe from separate glacial refugia, and, therefore, separate evolutionary events [77,78].

For insights into the geographical variation found in *Tshr*, the association of the SNP frequencies with the local climatic conditions was examined. Here, we showed that the genetic variation in the vole *Tshr* is better explained by the local photoperiod–temperature patterns than by only latitude. This may be caused by the temperature dependence of vegetation growth. In house mice, genes (but not the *Tshr*) that show signals of selection are also associated with the local average annual ambient temperature and are linked with clinal variation in the phenotypic aspects, such as body mass and metabolism [79,80]. Interestingly, the SNPs found in the thyroid hormone receptors, which are involved in the regulation of seasonal reproduction in the hypothalamus [35], significantly correlated with variation in the average annual temperature [80]. This suggests that the genomic evolution of seasonal adaptation in house mice and voles involves unique responses to genetic selection. Annual temperature patterns not only depend on latitude but also on longitude, altitude, and other regional climatic variables such as the Gulf Stream warming European Atlantic coastal regions. Critical photoperiods in pitcher-plant mosquitoes strongly correlated with altitude-corrected latitude (r = 0.96); however, this measurement did not integrate local temperature patterns [8,9,81]. Deriving the regional photoperiod–temperature ellipsoids may better account for such regional climatic differences than only latitude- or altitude-corrected latitude. We post hoc tested photoperiods at other temperature thresholds; however, this did not improve the results. Moreover, 6.6 °C is not an unreasonable temperature, since grass growth is initiated at 5–10 °C air temperature [3,57,58].

In addition, several SNPs correlated well with longitude and altitude (Figure 4G,J). Altitudinal gradients in the seasonal timing of breeding were observed in deer mice (*P. maniculatus*), with shorter breeding seasons occurring at high elevations [82]. The pCPP at which a temperature threshold for grass-growth initiation is reached can be deduced from local photoperiod–temperature patterns and is confirmed, here, to be a strong determinant for the distributional variation in the *Tshr* SNP frequency in Western European common vole populations (Figure 4M). Pairwise multilocus F_ST_ analysis revealed that populations that differ in pCPP also show a greater genetic distance in their *Tshr* haplotypes (Figure 3). These findings indicate that seasonality is likely to be a selective force for *Tshr* evolution in common voles and imply that *Tshr* is an important gene for the genetic adaptation of the photoperiodic response systems. Although the aim of the study was to establish the regional adaptation and selection, we cannot exclude the possibility that gender-specific selection is part of the mechanism driving the latitudinal selection for the alleles under study.

The observed genetic *Tshr* variation is unlikely to be caused by only isolation, with the possible exception of the Orkney Island populations, which are geographically isolated from each other and from mainland populations by the sea. Therefore, isolation and the related genetic drift may be more important evolutionary forces than natural selection in the Orkney Island populations. Interestingly, the same SNPs appear to be related to pCPP when the Orkney Island populations are excluded from the analysis. This indicates that the results in Western Europe are not dominated by the Orkney Island population’s data and that the observed distribution of the *Tshr* variation may be a sign of adaptive evolution, which is likely operating in response to the photoperiod.

In the Eastern European populations, none of the tested environmental proxies are good predictors for *Tshr* SNP frequencies (Figure 5). These results indicate that the *Tshr* in the Eastern European lineage is not linked to seasonal adaptation, as observed in the Western European lineage. Oceanic climates (Western Europe) are known for their small annual temperature amplitudes, while continental climates (Eastern Europe) are known for their large annual temperature amplitudes. These climatic differences may have led to the divergent evolutionary adaptation of the TSHR function, which may provide an explanation for the observed longitudinal separation in the genetic *Tshr* differentiation. Furthermore, we only sequenced a small part of one gene. Therefore, we cannot claim that this is the most important aspect of the regional adaptation of the photoperiodic neuroendocrine system. Another hypothesis is that genes other than *Tshr* are under selection for seasonal adaptation in vole populations.

The SNPs associated with local pCPP were all synonymous or intronic mutations. This suggests that these sites may be involved in regulatory rather than structural variation. Five intronic SNPs were strongly associated with pCPP in Western Europe (Figure 4), of which two (i.e., SNP-144 and -158) were strongly associated with altitude in Eastern Europe (Figure 5). Putative regulatory protein binding sites were predicted for the intronic region and revealed that intronic SNP-128, which strongly correlates with pCPP (Figure 4O), is located in a potential SP1 (specificity protein 1) binding site [83,84]. Interestingly, SNPs closely located to this enhancer region, such as SNP-158, are related to different environmental proxies in Eastern and Western Europe (Figure 4 and Figure 5 and Figure S2). It is tempting to speculate that variation in and around this SP1 binding site sequence may influence *Tshr* transcription. Furthermore, there is strong evidence that synonymous SNPs are not necessarily neutral, as they can alter mRNA expression, splicing, and structure, thus having downstream effects on protein expression [85,86]. Synonymous polymorphisms require different transfer RNAs (tRNA) to recruit the same amino acids and may cause codon bias. Synonymous tRNA vary strongly in frequency between species and tissues (i.e., codon bias) [87,88]. It is, therefore, possible that the observed synonymous mutations in the *TSHR* may alter translation efficiency within a species and tissue by changing the elongation rate [89]. A reduced elongation rate may, therefore, result in a lower protein abundance. Hence, synonymous SNPs in the *Tshr* gene could result in an altered receptor abundance, a changed sensitivity to TSH, and a modified photoperiodic response. It is, therefore, conceivable that synonymous SNPs in the *Tshr* gene are subject to natural selection and reflect local geographical adaptation. TSHR plays a pivotal role in photoperiodic response not only in the pars tuberalis [90] but also in the thyroid hormone metabolism in the thyroid gland [91]. The duration of the melatonin signal encodes daylength and acts on the melatonin receptors in the pars tuberalis, causing the suppression of TSHβ release [90]. Interestingly, the TSH produced by the pars tuberalis (acting on the hypothalamus to regulate seasonality) and the TSH produced by the pars distalis (stimulating the thyroid gland to produce thyroid hormones) prevent functional crosstalk by tissue-specific glycosylation [91]. The tissue-specific functions of TSHR may benefit from the genetic adaptation in photoperiodism through synonymous SNPs, since tissue-specific tRNA expression, which has been demonstrated in human and mouse tissues [88,92], may perhaps lead to altered TSHR function in the pars tuberalis but not in the thyroid gland.

The photoperiodic regulation of the reproductive system in deer mice was shown to vary with latitude, with weaker photoperiodic responses in animals originating from lower latitudes [20]. Moreover, photoperiodic sensitivity in pitcher-plant mosquitoes correlated with global warming, indicating the importance of the season-length driving evolution (genetic change) of photoperiodism during the recent, rapid climate change [93,94,95,96]. Our findings confirm that the *Tshr* gene is under selection, which was previously reported in chicken domestication in relation to photoperiodic responsiveness [39,42]. Future studies should determine whether the SNPs identified as a seasonal-timing-dependent genetic variation in the vole *Tshr* can indeed alter genetically based photoperiodic responses. Such an approach should confirm whether habitat-specific photoperiodic responses are indeed regulated by means of functional TSHR adaptation. In vole populations with later onsets of reproduction and shorter breeding seasons [97], our results predict lower concentrations in the tanycytes of *Tshr* or the lower TSH-binding affinities of *Tshr* haplotypes.

Optimal timing of reproduction, enhancing energetically demanding pregnancy, and parental care are necessary to maximize fitness in temperate and northern seasonal environments. *Tshr* is an essential gene in the pathway programming seasonal reproduction in mammals. Herein, we show how the onset of the favorable season, over a wide geographical range of the common vole, *M. arvalis*, explains much of the genetic variation in the TSH binding site, hinge region, and transmembrane domain of *Tshr* in Western Europe but not Eastern Europe. Yet vole populations thrive in both regions. We, therefore, conclude that different genetic mechanisms have been important in enabling vole populations to exploit geographically distinct regions. Such distinctions, regarding how the genetic underpinnings of seasonal timing have evolved over climatic gradients in nature, are important in predicting how animals will adapt to new seasonal environments during the ongoing, rapid climate change [98].

## Data Availability

The raw sequence reads and metadata will be deposited in a public repository (FigShare, https://doi.org/10.6084/m9.figshare.14695356.v1 (accessed on 7 December 2022)) after acceptance.

## Supplementary Materials

The following supporting information can be downloaded at: https://www.mdpi.com/article/10.3390/genes14020292/s1. Figure S1: *Tshr* mutations in Western and Eastern European common vole populations; Figure S2: A distance-based phylogenetic tree for the sequenced *Tshr* region, inferred with the neighbor-joining method; Figure S3: Pairwise linkage disequilibrium heatmaps; Table S1: List of locations where *M. arvalis* specimens were obtained; Table S2: Primer sequences; Table S3: Thermal cycling conditions for PCR; Table S4: Nucleotide and predicted amino acid sequences; Table S5: Statistical output for linear models relating pairwise distances of allele frequencies to pairwise differences in environmental proxies (geographical distance, latitude, longitude, altitude, and pCPP) in Eastern and Western Europe; Table S6: Statistical output for linear models relating pairwise distances of allele frequencies to pairwise differences in environmental proxies (geographical distance, latitude, longitude, altitude, and pCPP) in Western Europe; Table S7: Statistical output for linear models relating pairwise distances of allele frequencies to pairwise differences in environmental proxies (geographical distance, latitude, longitude, altitude, and pCPP) in Eastern Europe.
