# Supplementary material for: Seasonal Adaptation: Geographic Photoperiod–Temperature Patterns Explain Genetic Variation in the Common Vole Tsh Receptor
Source: Genes (Basel). 2023 Jan 22;14(2):292. doi: 10.3390/genes14020292 (PMC9957289; doi:10.3390/genes14020292)
Supplement: Supplementary file 1 [file genes-14-00292-s001.zip › genes-2114717-supplementary-Jan.29.pdf]

genes MDPI

Supplementary Information for

Seasonal Adaptation: Geographic Photoperiod-Temperature Patterns Explain Genetic Variation in the Common Vole Tsh Receptor

Laura van Rosmalen [1,†], Robin Schepers [1], Wensi Hao [1], Anna S. Przybylska-Piech [1,‡], Jeremy S. Herman [2], Joanna Stojak [3,4], Jan M. Wójcik [3], Louis van de Zande [5], Jeremy B. Searle [6,*] and Roelof A. Hut [1]

This PDF file includes:

Figures S1 to S3
Tables S1 to S7

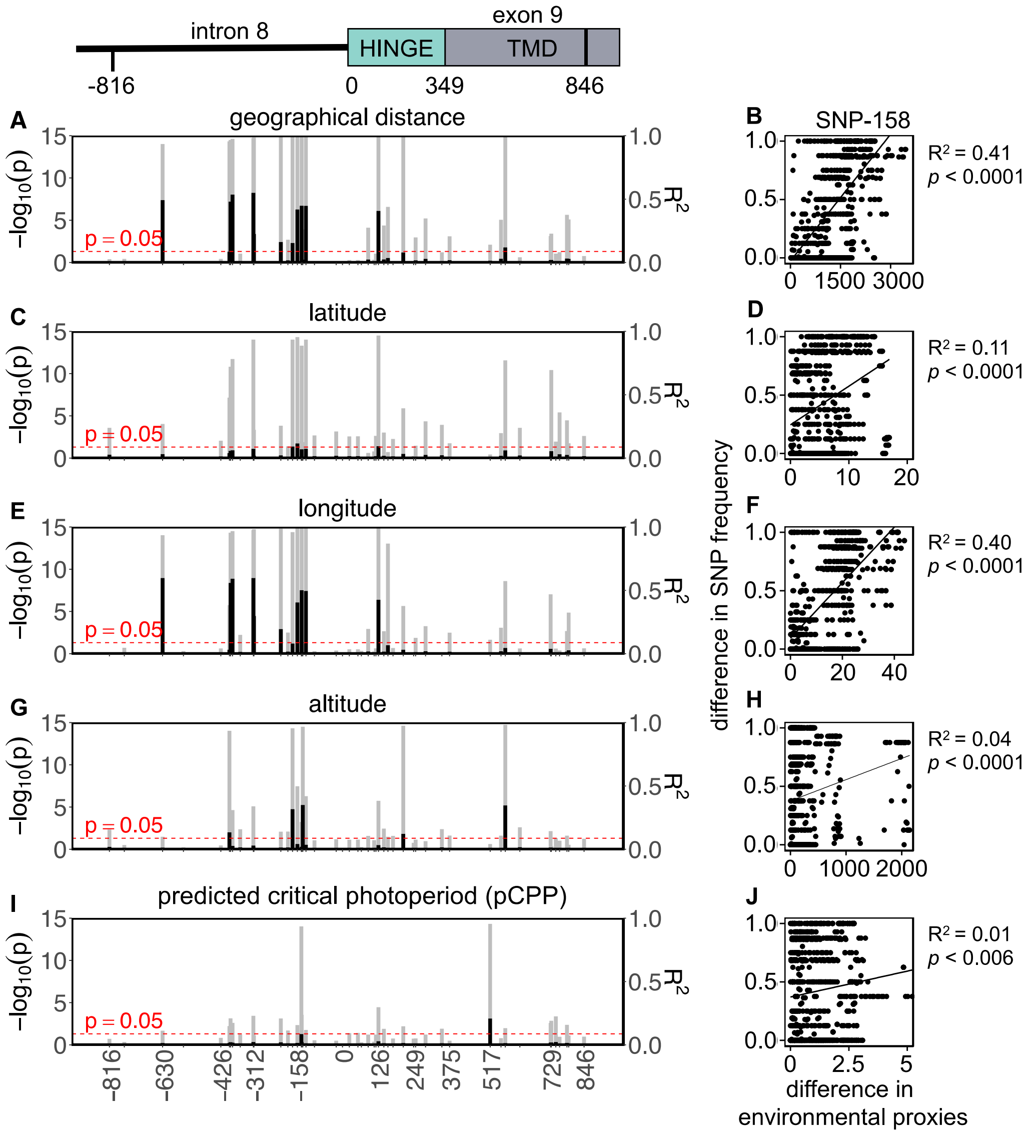

Figure S1. *Tshr* mutations in Western and Eastern European common vole populations. Manhattan-type plots (-$\log_{10}(p)$) for the sequenced *Tshr* region for (A) geographical distance, (C) latitude, (E) longitude, (G) altitude and (I) predicted critical photoperiod (pCPP). Grey bars indicate Benjamini-Hochberg adjusted p-values, black bars indicate R^2-values. SNPs that meet the threshold for

significant correlations ($p < 0.05$) cross the red dashed line. Pairwise difference in SNP frequency for one representative mutations (SNP-158) related to (B) pairwise geographical distance, (D) pairwise latitudinal difference, (F) pairwise longitudinal difference, (H) pairwise altitudinal difference and (J) pairwise difference in pCPP. Significant correlations are indicated by linear regression lines. All statistic results of linear models for SNP frequency related to environmental proxies can be found in Table S5.

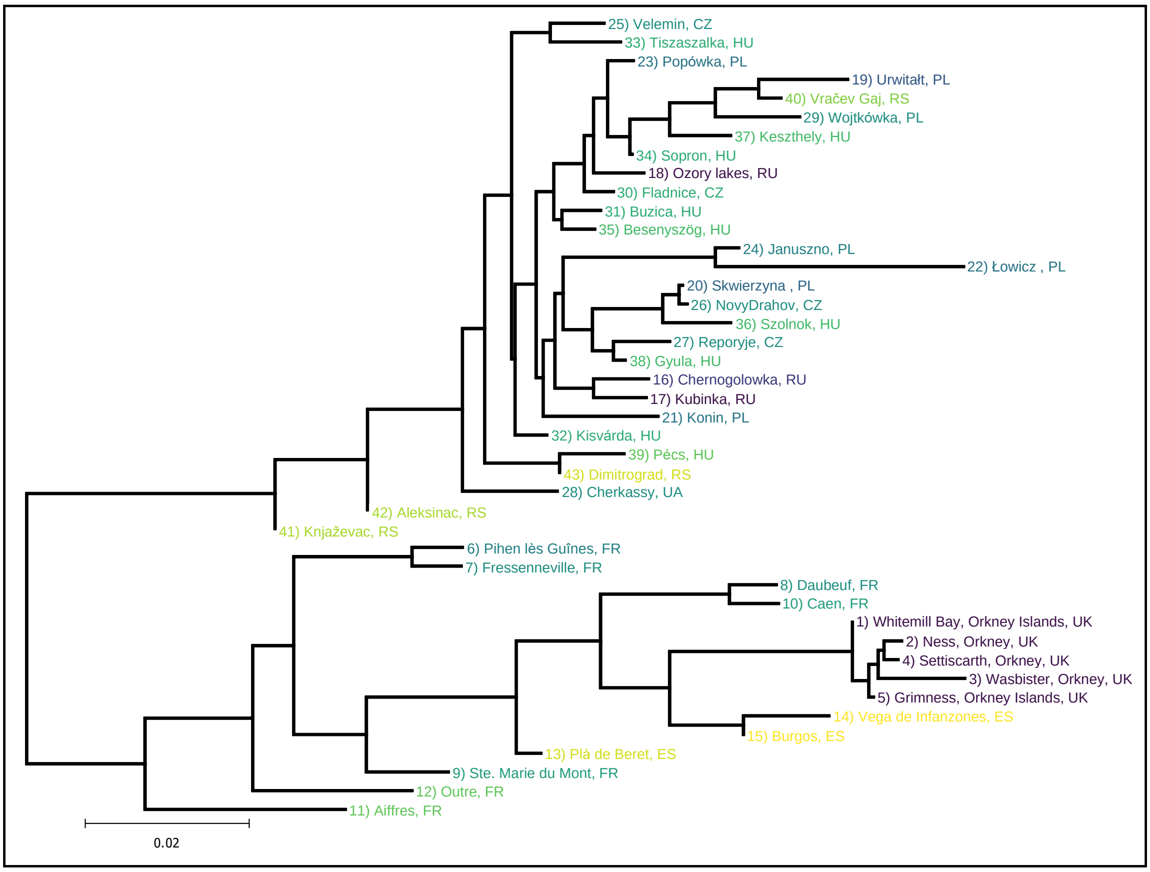

Figure S2. A distance-based phylogenetic tree for the sequenced Tshr region, inferred with the neighbor-joining method. Populations are labelled using region names (see Fig. 1 and Table S1). Colors indicate latitude as in Figure 1A, ranging from 42°N (yellow) to 59°N (purple).

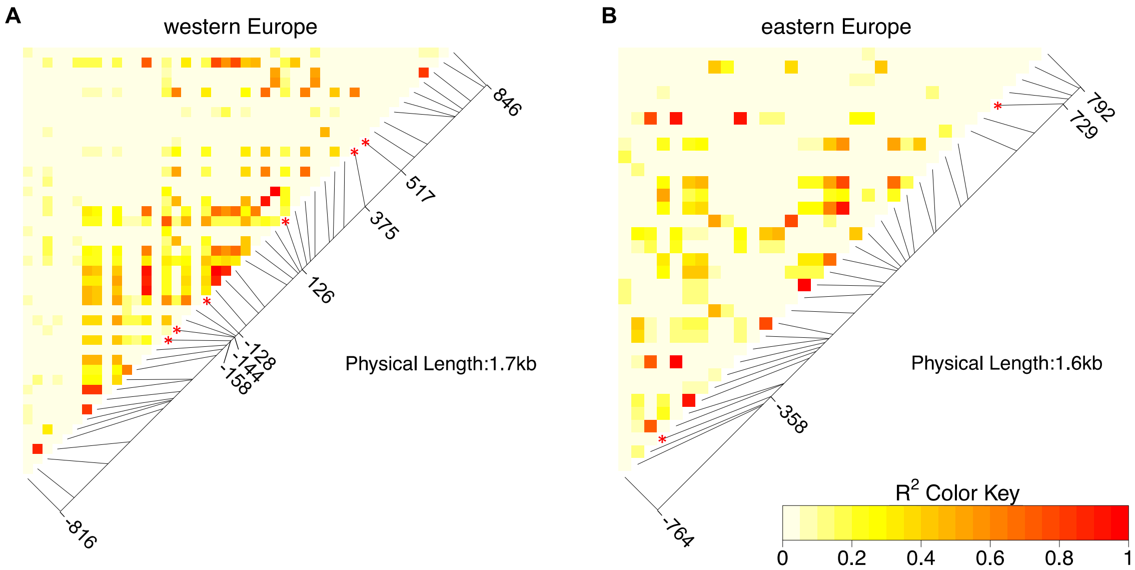

Figure S3. Pairwise linkage disequilibrium heatmaps. Patterns of linkage disequilibrium between SNPs within the sequenced Tshr region for (A) Western and (B) Eastern European samples. Colors indicate R2-values for pairwise correlations, varying between 0 (white) and 1 (red). Mutations with SNP frequencies that significantly correlate with local temperature thresholds are marked with red asterisks.

Table S1. List of locations where M. arvalis specimens were obtained (country codes: CZ - Czech Republic, ES - Spain, FR - France, HU - Hungary, PL - Poland, RS – Serbia, RU – Russia, UA - Ukraine, UK - Great Britain), pCPP – predicted critical photoperiod.

Map reference (Fig1A)	Location	Year	Latitude	Longitude	Altitude (m)	n	pCPP (h)	tissue	Provided by
1	Whitemill Bay, Orkney Islands, UK	2006	59.30	-2.55	3	11	15.03	toe	J.B. Searle, J.S. Herman
2	Ness, Orkney Islands, UK	2006	59.23	-2.87	-1	11	15.03	toe	J.B. Searle, J.S. Herman
3	Wasbister, Orkney Islands, UK	2006	59.18	-3.06	20	9	15.03	toe	J.B. Searle, J.S. Herman
4	Settiscarth, Orkney Islands, UK	2006	59.05	-3.12	104	11	15.03	toe	J.B. Searle, J.S. Herman
5	Grimness, Orkney Islands, UK	2006	58.82	-2.92	75	11	15.03	toe	J.B. Searle, J.S. Herman
6	Pihen lès Guînes, FR	2007	50.87	1.79	51	21	12.33	toe	J.B. Searle, J.S. Herman
7	Fressenneville, FR	2007	50.07	1.58	98	12	12.48	toe	J.B. Searle, J.S. Herman
8	Daubeuf, FR	2007	49.78	0.07	103	12	12.19	toe	J.B. Searle, J.S. Herman
9	Ste. Marie du Mont, FR	2007	49.37	-1.23	138	11	12.59	toe	J.B. Searle, J.S. Herman
10	Caen, FR	2007	49.26	-0.45	67	12	12.28	toe	J.B. Searle, J.S. Herman
11	Aiffres, FR	2002	46.27	-0.41	10	11	10.19	toe	J.B. Searle, J.S. Herman
12	Outre, FR	2005	46.08	3.17	330	12	12.40	toe	J.B. Searle, J.S. Herman
13	Plà de Beret, ES	2001	42.72	0.84	2146	7	13.50	ear	J.B. Searle, J.S. Herman

14	Vega de Infanzones, ES	2007	42.48	-5.65	914	7	13.21	toe	J.B. Searle, J.S. Herman
15	Burgos, ES	2007	42.36	-3.70	891	11	13.03	toe	J.B. Searle, J.S. Herman
16	Czernogolowka, RU	2014	56.01	38.39	160	4	15.40	leg	J. Stojak, J.M. Wójcik
17	Kubinka, RU	2014	55.28	36.40	200	3	15.21	leg	J. Stojak, J.M. Wójcik
18	Ozory Lakes, RU	2014	54.51	38.33	200	4	15.12	leg	J. Stojak, J.M. Wójcik
19	Urwitałt, PL	2014	53.81	21.64	111	4	14.30	leg	J. Stojak, J.M. Wójcik
20	Skwierzyna, PL	2014	52.60	15.51	28	4	13.43	leg	J. Stojak, J.M. Wójcik
21	Konin, PL	2014	52.22	18.25	104	4	13.91	leg	J. Stojak, J.M. Wójcik
22	Łowicz, PL	2014	52.06	19.56	80	3	13.97	leg	J. Stojak, J.M. Wójcik
23	Popówka, PL	2014	52.04	23.26	141	3	14.10	leg	J. Stojak, J.M. Wójcik
24	Januszno, PL	2014	51.29	21.30	160	3	13.60	leg	J. Stojak, J.M. Wójcik
25	Velemin, CZ	2011	50.32	13.58	244	4	13.23	leg	J. Stojak, J.M. Wójcik
26	NovyDrahov, CZ	2011	50.14	12.39	440	4	14.17	leg	J. Stojak, J.M. Wójcik
27	Reporyje, CZ	2011	50.01	14.17	412	4	13.76	muscle	J. Stojak, J.M. Wójcik
28	Cherkassy, UA	2012	49.87	31.43	177	2	13.71	leg	J. Stojak, J.M. Wójcik
29	Wojtkówka, PL	2014	49.56	22.56	420	4	13.38	leg	J. Stojak, J.M. Wójcik
30	Fladnice, CZ	2011	48.48	15.59	360	5	12.97	leg	J. Stojak, J.M. Wójcik
31	Buzica, HU	2013	48.32	21.04	216	4	12.92	muscle	J. Stojak, J.M. Wójcik
32	Kisvárda, HU	2013	48.22	22.08	103	4	12.76	leg	J. Stojak, J.M. Wójcik
33	Tiszaszalka, HU	2013	48.19	22.31	106	5	12.76	leg	J. Stojak, J.M. Wójcik
34	Sopron, HU	2013	47.68	16.58	217	5	12.90	leg	J. Stojak, J.M. Wójcik
35	Besenyszög, HU	2013	47.30	20.26	85	4	12.31	leg	J. Stojak, J.M. Wójcik
36	Szolnok, HU	2013	47.16	20.18	97	4	12.31	leg	J. Stojak, J.M. Wójcik
37	Keszthely, HU	2013	46.77	17.25	116	4	12.82	leg	J. Stojak, J.M. Wójcik
38	Gyula, HU	2013	46.65	21.28	87	4	12.58	leg	J. Stojak, J.M. Wójcik
39	Pécs, HU	2013	46.07	18.23	152	4	12.49	leg	J. Stojak, J.M. Wójcik
40	Vračev Gaj, RS	2013	44.88	21.32	76	4	12.39	leg	J. Stojak, J.M. Wójcik
41	Knjaževac, RS	2013	43.57	22.25	220	4	12.69	leg	J. Stojak, J.M. Wójcik

42	Aleksinac, RS	2013	43.54	21.72	271	4	12.69	muscl e	J. Stojak, J.M. Wójcik
43	Dimitrovgrad, RS	2013	43.01	22.77	560	4	12.77	leg	J. Stojak, J.M. Wójcik

Table S2. Primer sequences used for amplification (PCR) and Sanger sequencing of the *Tshr* gene of the common vole.

Gene	Forward primer ('5-'3)	Reverse primer ('5-'3)	Product size (bp)
Tshr intron8	GGTGGAAAAGATGGCTCGAA	GGAGCCCCTTAAACTCTGGG	1102
Tshr exon9-1	TTGGAGTGATTTCTGACTAGG	TATCGCGAGGGTTGTACT	1208

Table S3. Thermal cycling conditions for PCR.

PCR step	T (°C)	Duration (seconds)	Cycles
Intial denaturation	95	120	
Denaturation	95	30	35
Annealing	60	30	
Extension	72	60	
Final extension	72	420	
	4	<24h	

Table S4. Nucleotide and predicted amino acid sequence of the end of intron 8 and the beginning of exon 9 of the common vole *Tshr*. SNPs shown in red.

```
                 GTATCATAGAGGATTTACCAATGTTTGCTGAGAAAATACTAAGCT -1014

ACACAGAAGTAGAATTTACCTGAAAAAGTACTGCCTCCAACACAACTCTAAGACAAATAG -960

CATGCTAACCAAACCTAGAAAGTATATAAAATAAGGAAGTCAGAGTTATACTAGCACAGT -900

AACTTAGAATTCAGACTCCCTCAGGRCATGTTTCTTTAGGACTGGCTCATAGGGAGCCCC -840

TTTCCTCATCAGAGGAAYGCAAAGGGAAAATGGCCCTGCTGCCAGCTGTTCAGACAGCAA -780

ACCTTGTTTGTTGAACAGAGAGCTGCAATCTAAAGATAACCAAAAACTGCTGCTTCATTT -720

TAGTTCTAGAAAACTCACCTAATGAGTCGGGNGCACGCCTTTAATCCCAGCACTTGGGAG -660

GCAGAGGCAGGTGGATCTCTGTGAGTTTGAGGCCAGCCTGATCTMCAAGAGCTAGTTCCA -600

GGACAGGCTCCAAAGCTACAGAGAAACCCTGTCTTGAAAAAAAAAAGAAAAGAAAAGAAA -540

GAAGGAAGGAAGGAAGGAAGGAAGGAAGGAAGGAAGGAAAAGAAAGCCCACCTAAYTGCT -480

CAGAAAATGTAGTTGGGATGGGAGMTTTMAAAGCCWACTCAATGCATATGTAAGGTTACC -420

ATKTTAGAACTCAAGACTCCGAGCTGTAGACAATTTCTGGTCATGGCCRGRATCCCATAG -360

GCTTAACCAGAATCTGGATGCATGCTTTTATTAAAGTGTTCTTGTTGATCTTTGAAGTGC -300

TTGGAGTGATTTCTGACTAGGAAGRTTTAATTCTGCCAGTGAAACTCTAYGCCATGCTGT -240

GGTGCWTGCAACATAACAAAGTSAATGTTTTAAATGSYGTASCAGAGGAGGGYTCATCCA -180

GATCATGAAGAATCAGTAGCACYAAAACATCAGAGGAAGTAACTTGTGTATCTTCCCAGA -120

GTTTAAGGGGCTCCMAGGTGGACTTTAAGCAAGAGGTGRCTCCTATTTGTTGCCTTACAG -60

ATCCTGGAGTCCTTGATGTGTAAYGAGAGTAGCATCCGGAATCTGCGCCAGAGRAAATCA 60
 I  L  E  S  L  M  C  N  E  S  S  I  R  N  L  R  Q  R  K  S  20

GTGAAYGTGCTGAGAGGTCCCCTCTACCAGGAGTACGAGGAAGATCTGAGYGACAAYGGT 120
 V  N  V  L  R  G  P  L  Y  Q  E  Y  E  E  D  L  S  D  N  G  40

GTTGGRTACAAACAAAACCCCAAGYTCCAGGAGAGCTCRGGCAACTCTCATTACTAYGTC 180
 V  G  Y  K  Q  N  P  K F/L Q  E  S  S  G  N  S  H  Y  Y  V  60

TTCTTTGAAGAACAAGAGGATGAGATCCTTGGYTTCGGCCAAGAGCTCAAAAATCCTCAG 240
 F  F  E  E  Q  E  D  E  I  L  G  F  G  Q  E  L  K  N  P  Q  80

GAAGAGACKCTGCTARCCTTTGACAGCCACTATGACTACACTGTGTGTGGGNACAATGAA 300
 E  E  T  L  L T/A F  D  S  H  Y  D  Y  T  V  C  G DNH N  E  100

GACATGGTGTGCACCCCCAAGTCGGATGAGTTTAACCCCTGTGAAGAYATCATGGGTTAC 360
 D  M  V  C  T  P  K  S  D  E  F  N  P  C  E  D  I  M  G  Y  120

AAGTTCCTGAGAATMGTGGTGTGGTTTGTCAGTCTGCTGGCTCTCCTGGGCAACATCTTT 420
 K  F  L  R  I  V  V  W  F  V  S  L  L  A  L  L  G  N  I  F  140

GTCCTGTTTATTCTCCTCACCAGCCACTACAAACTGACCGTGCCTCGCTTTCTTATGTGT 480
 V  L  F  I  L  L  T  S  H  Y  K  L  T  V  P  R  F  L  M  C  160

AACTTGGCCTTTGCGGACTTCTGCATGGGGGTATACCTGCTGCTCATTGCCTCTGTGGAC 540
 N  L  A  F  A  D  F  C  M  G  V  Y  L  L  L  I  A  S  V  D  180

CTGTACACACAGTCRGAGTACTACAACCAYGCCATCGACTGGCAGACTGGCCCTGGGTGC 600
 L  Y  T  Q  S  E  Y  Y  N  H  A  I  D  W  Q  T  G  P  G  C  200

AACACGGCTGGCTTCTTCACYGTTTTTGCCAGCGAACTATCCGTGTACACGCTAACAGTC 660
 N  T  A  G  F  F  T  V  F  A  S  E  L  S  V  Y  T  L  T  V  220

ATCACCCTGGAGCGATGGTATGCCATCACCTTCGCCATGCGCCTGGACAGGAAGATCCGC 720
 I  T  L  E  R  W  Y  A  I  T  F  A  M  R  L  D  R  K  I  R  240

CTGCGGCAYGYGTACACCATCATGGCKGGGGGCTGGGTTKCCTGCTTCCTCCTGGCCCTG 780
 L  R  H A/V Y  T  I  M  A  G  G  W  V S/A C  F  L  L  A  L  260

CTCCCYTTGGTRGGAATAAGCAGCTACGCCAAGGTCAGCATCTGCCTGCCGATGGACACT 840
 L  P  L  V  G  I  S  S  Y  A  K  V  S  I  C  L  P  M  D  T  280

GACACNCCT                                                    850
 D  T  P                                                     20
```

Table S5. Statistical output for linear models relating pairwise distances of allele frequencies to pairwise difference in environmental proxies (geographical distance, latitude, longitude, altitude and pCPP) in Eastern and Western Europe. SNP loc.: location of SNP relative to intron 8 – exon 9 boundary. SNP: changed nucleotides. Amino acid: changed amino acids are indicated with an asterisk. FST: F-statistic reflecting overall degree of genetic differentiation. pCPP: predicted critical photoperiod. p.adj: Benjamini-Hochberg adjusted p-value. Significant correlations are marked with an asterisk (p<0.05).

WESTERN AND EASTERN EUROPE							geographical distance				latitude				longitude				altitude				pCPP			
#	SNP loc.	SNP		amino acid		F_{ST}	R^2	$F_{1,901}$	*p*	*p.adj*	R^2	$F_{1,901}$	*p*	*p.adj*	R^2	$F_{1,901}$	*p*	*p.adj*	R^2	$F_{1,901}$	*p*	*p.adj*	R^2	$F_{1,901}$	*p*	*p.adj*
-22	-816	A	G			0.542	0.00	0.77	3.79E-01	4.65E-01	0.02	14.81	1.31E-04	3.05E-04*	0.00	0.07	7.93E-01	8.27E-01	0.01	9.67	1.96E-03	5.32E-03*	0.00	1.85	1.75E-01	2.20E-01
-21	-764	C	T			0.242	0.00	0.91	3.41E-01	4.40E-01	0.00	0.07	7.86E-01	8.37E-01	0.00	2.11	1.47E-01	2.32E-01	0.00	0.99	3.21E-01	3.66E-01	0.00	0.53	4.66E-01	5.19E-01
-20	-630	C	A/T			0.944	0.49	597.80	2.20E-16	1.08E-14*	0.03	17.30	3.64E-05	1.05E-04*	0.59	913.80	2.20E-16	1.08E-14*	0.01	5.37	2.09E-02	3.79E-02*	0.01	6.55	1.08E-02	2.39E-02*
-19	-557	A	C			0.097	0.00	0.01	9.14E-01	9.33E-01	0.00	0.03	8.68E-01	8.86E-01	0.00	0.43	5.11E-01	5.69E-01	0.00	0.17	6.84E-01	6.84E-01	0.00	0.08	7.74E-01	8.24E-01
-18	-426	T	C			0.818	0.00	0.88	3.48E-01	4.37E-01	0.01	7.42	6.57E-03	1.01E-02*	0.00	1.71	1.91E-01	2.76E-01	0.00	0.81	3.68E-01	4.10E-01	0.00	2.47	1.16E-01	1.67E-01
-17	-396	C	A			0.082	0.08	79.01	2.20E-16	5.39E-15*	0.03	32.28	1.80E-08	8.04E-08*	0.03	25.18	6.28E-07	2.05E-06*	0.13	134.10	2.20E-16	1.08E-14*	0.01	9.68	1.92E-03	6.73E-03*
-16	-392	C	A			0.646	0.48	818.50	2.20E-16	3.59E-15*	0.05	49.91	3.21E-12	1.75E-11*	0.55	1121.00	2.20E-16	5.39E-15*	0.01	11.39	7.70E-04	2.69E-03*	0.02	14.95	1.18E-04	8.29E-04*
-15	-385	T	A			0.766	0.53	1022.00	2.20E-16	2.70E-15*	0.06	54.81	3.05E-13	2.14E-12*	0.59	1281.00	2.20E-16	3.59E-15*	0.02	20.97	5.33E-06	2.61E-05*	0.01	11.90	5.88E-04	2.88E-03*
-14	-358	G	T			0.503	0.00	3.38	6.64E-02	1.02E-01	0.00	0.02	8.98E-01	8.98E-01	0.01	8.57	3.50E-03	6.86E-03*	0.01	9.99	1.63E-03	4.98E-03*	0.00	4.39	3.64E-02	6.85E-02
-13	-312	A	G			0.782	0.54	1078.00	2.20E-16	2.16E-15*	0.07	66.57	1.13E-15	1.11E-14*	0.59	1307.00	2.20E-16	2.70E-15*	0.03	23.18	1.73E-06	9.40E-06*	0.02	16.56	5.13E-05	4.19E-04*
-12	-310	A	G			0.621	0.22	255.50	2.20E-16	1.80E-15*	0.01	13.65	2.34E-04	5.21E-04*	0.29	374.10	2.20E-16	2.16E-15*	0.00	0.42	5.19E-01	5.52E-01	0.00	3.81	5.13E-02	8.38E-02
-11	-216	G	A			0.648	0.16	167.60	2.20E-16	1.54E-15*	0.02	16.05	6.66E-05	1.72E-04*	0.19	210.90	2.20E-16	1.80E-15*	0.01	8.27	4.14E-03	9.65E-03*	0.02	14.66	1.38E-04	8.43E-04*
-10	-191	T	C			0.385	0.01	10.36	1.44E-01	2.34E-03*	0.01	4.33	3.79E-02	4.76E-02*	0.01	9.81	1.80E-03	3.68E-03*	0.01	8.46	3.74E-03	9.15E-03*	0.01	6.83	9.11E-03	2.23E-02*
-9	-175	A	T			0.306	0.15	143.60	2.20E-16	1.35E-15*	0.09	77.58	2.20E-16	1.08E-14*	0.08	66.95	1.06E-15	4.74E-15*	0.31	372.20	2.20E-16	5.39E-15*	0.00	0.08	7.75E-01	8.08E-01
-8	-158	G	C			0.644	0.41	637.10	2.20E-16	1.20E-15*	0.11	112.90	2.20E-16	5.39E-15*	0.40	601.70	2.20E-16	1.54E-15*	0.04	34.90	4.92E-09	4.02E-08*	0.01	10.45	1.27E-03	5.19E-03*
-7	-144	G	C			0.225	0.00	2.14	1.44E-01	2.01E-01	0.01	7.10	7.84E-03	1.16E-02*	0.00	1.19	2.76E-01	3.56E-01	0.00	0.26	6.12E-01	6.24E-01	0.08	79.79	2.20E-16	1.08E-14*
-6	-143	T	C			0.655	0.44	717.50	2.20E-16	1.08E-15*	0.07	62.75	6.88E-15	5.62E-14*	0.50	889.00	2.20E-16	1.35E-15*	0.02	14.48	1.51E-04	6.74E-04*	0.02	17.88	2.59E-05	3.17E-04*
-5	-139	G	C			0.186	0.02	16.14	6.36E-05	1.42E-04*	0.01	6.52	1.08E-02	1.51E-02*	0.00	3.97	4.68E-02	7.90E-02	0.35	476.20	2.20E-16	3.59E-15*	0.00	4.01	4.56E-02	7.71E-02
-4	-128	T	C			0.670	0.44	714.50	2.20E-16	9.80E-16*	0.07	67.09	8.84E-16	1.08E-14*	0.49	867.20	2.20E-16	1.20E-15*	0.03	29.18	8.43E-08	5.90E-07*	0.01	7.17	7.54E-03	1.94E-02*
-3	-98	C	T			0.000	0.00	0.03	8.65E-01	9.21E-01	0.01	10.45	1.27E-03	2.40E-03*	0.00	0.39	5.34E-01	5.81E-01	0.00	3.30	6.98E-02	9.77E-02	0.00	1.21	2.71E-01	3.32E-01
-2	-46	A	C			0.000	0.01	10.41	1.30E-03	2.36E-03*	0.02	22.80	2.09E-06	7.33E-06*	0.00	2.22	1.37E-01	2.23E-01	0.01	12.30	4.75E-04	1.94E-03*	0.01	6.62	1.03E-02	2.39E-02*
-1	-22	G	A			0.000	0.00	0.00	9.48E-01	9.48E-01	0.01	12.73	3.78E-04	8.06E-04*	0.00	0.66	4.15E-01	4.85E-01	0.00	3.93	4.79E-02	7.56E-02	0.00	1.03	3.11E-01	3.72E-01
1	24	C	T	N	N	0.388	0.00	0.43	5.11E-01	5.97E-01	0.01	9.76	1.85E-03	3.12E-03*	0.00	1.15	2.83E-01	3.56E-01	0.00	3.61	5.77E-02	8.57E-02	0.01	5.29	2.17E-02	4.62E-02*
2	54	A	G	R	R	0.426	0.00	0.43	5.11E-01	5.83E-01	0.01	9.76	1.85E-03	3.02E-03*	0.00	1.15	2.83E-01	3.47E-01	0.00	3.61	5.77E-02	8.32E-02	0.01	5.29	2.17E-02	4.43E-02*
3	66	T	C	N	N	0.212	0.00	0.17	6.82E-01	7.59E-01	0.00	0.03	8.52E-01	8.88E-01	0.00	0.52	4.71E-01	5.36E-01	0.00	0.29	5.89E-01	6.14E-01	0.00	0.40	5.29E-01	5.76E-01
4	90	G	A	Q	Q	0.000	0.02	17.16	3.76E-05	8.77E-05*	0.00	1.37	2.43E-01	2.83E-01	0.01	12.01	5.53E-04	1.36E-03*	0.01	5.89	1.54E-02	3.02E-02*	0.00	4.26	3.92E-02	6.86E-02
5	111	T	C	S	S	0.336	0.00	0.56	4.53E-01	5.42E-01	0.00	2.06	1.52E-01	1.86E-01	0.00	0.27	6.04E-01	6.43E-01	0.00	3.26	7.14E-02	9.71E-02*	0.00	0.64	4.25E-01	4.84E-01
6	117	C	G/T	G	G	0.000	0.00	3.28	7.06E-02	1.02E-01	0.01	10.08	1.55E-03	2.72E-03*	0.00	1.68	1.96E-01	2.67E-01	0.00	1.18	2.78E-01	3.32E-01	0.00	4.32	3.81E-02	6.91E-02
7	126	A	G	G	G	0.561	0.40	606.10	2.20E-16	8.98E-16*	0.09	88.26	2.20E-16	3.59E-15*	0.42	655.50	2.20E-16	1.08E-15*	0.03	26.37	3.46E-07	2.12E-06*	0.02	22.50	2.45E-06	4.00E-05*
8	145	T	C	F	L*	0.647	0.02	18.39	2.00E-05	4.89E-05*	0.01	12.07	5.38E-04	1.10E-03*	0.01	10.65	1.14E-03	2.54E-03*	0.01	10.46	1.26E-03	4.13E-03*	0.01	7.80	5.34E-03	1.45E-02*
9	159	G	A	S	S	0.637	0.03	29.01	9.22E-08	3.01E-07*	0.00	1.73	1.89E-01	2.26E-01	0.06	59.93	2.64E-14	1.08E-13*	0.01	5.28	2.18E-02	3.81E-02*	0.00	0.07	7.93E-01	8.10E-01
10	177	C	T	Y	Y	0.174	0.00	0.02	8.76E-01	9.13E-01	0.01	8.34	3.98E-03	6.28E-03*	0.00	1.98	1.60E-01	2.44E-01	0.01	5.83	1.60E-02	3.02E-02*	0.00	2.05	1.53E-01	2.02E-01
11	213	T	C	N	N	0.000	0.07	72.74	2.20E-16	8.29E-16*	0.03	26.3	3.57E-07	1.46E-06*	0.03	24.59	8.47E-07	2.60E-06*	0.12	119.60	2.20E-16	2.70E-15*	0.01	9.39	2.24E-03	7.32E-03*
12	249	G	T	T	T	0.299	0.00	0.09	7.64E-01	8.32E-01	0.00	0.28	5.95E-01	6.48E-01	0.00	0.01	9.15E-01	9.34E-01	0.00	2.02	1.56E-01	1.96E-01	0.00	1.97	1.61E-01	2.07E-01
13	256	G	A	A	T*	0.962	0.01	11.66	6.69E-04	1.26E-03*	0.01	4.72	3.00E-02	3.87E-02*	0.01	7.03	8.17E-03	1.54E-02*	0.00	3.74	5.35E-02	8.20E-02	0.00	2.13	1.45E-01	1.97E-01
14	291	G	A/C	D	N/H*	0.000	0.02	22.52	2.42E-06	6.98E-06*	0.02	18.75	1.66E-05	5.09E-05*	0.01	13.62	2.37E-04	6.46E-04*	0.00	2.75	9.79E-02	1.26E-01	0.00	4.45	3.51E-02	6.88E-02
15	348	C	T	D	D	0.445	0.00	4.09	4.34E-02	7.10E-02	0.02	16.66	4.87E-05	1.33E-04*	0.00	0.00	9.97E-01	9.97E-01	0.01	9.98	1.63E-03	4.70E-03*	0.01	7.99	4.82E-03	1.39E-02*
16	375	C	A	I	I	0.279	0.01	12.32	4.72E-04	9.24E-04*	0.01	5.93	1.51E-02	2.00E-02*	0.01	9.83	1.77E-03	3.77E-03*	0.01	6.22	1.28E-02	2.73E-02*	0.00	1.01	3.15E-01	3.67E-01
17	517	C	T/G/A	L	L	0.000	0.01	7.82	5.30E-03	8.95E-03*	0.00	0.77	3.80E-01	4.33E-01	0.01	6.07	1.40E-02	2.54E-02*	0.00	1.49	2.23E-01	2.74E-01	0.20	219.40	2.20E-16	5.39E-15*
18	555	G	A	S	S	0.502	0.02	21.56	3.97E-06	1.02E-05*	0.01	11.72	6.47E-04	1.27E-03*	0.01	12.72	3.81E-04	9.83E-04*	0.01	6.23	1.27E-02	2.84E-02*	0.00	2.26	1.34E-01	1.87E-01
19	570	C	T	H	H	0.000	0.11	116.60	2.20E-16	7.70E-16*	0.06	53.78	4.99E-13	3.05E-12*	0.04	38.63	7.81E-10	2.94E-09*	0.34	468.50	2.20E-16	2.16E-15*	0.01	8.35	3.95E-03	1.21E-02*
20	621	C	T	T	T	0.000	0.00	1.59	2.08E-01	2.76E-01	0.02	14.86	1.24E-04	3.04E-04*	0.00	1.75	1.86E-01	2.76E-01	0.01	11.41	7.61E-04	2.87E-03*	0.00	0.05	8.19E-01	8.19E-01
21	729	C	T	H	H	0.345	0.01	12.53	4.20E-04	8.58E-04*	0.00	0.33	5.66E-01	6.30E-01	0.03	31.22	3.05E-08	1.07E-07*	0.01	8.75	3.17E-03	8.18E-03*	0.01	11.71	6.51E-04	2.90E-03*
22	731	C	T	A	V*	0.660	0.02	13.88	2.07E-04	4.41E-04*	0.05	47.80	8.94E-12	4.38E-11*	0.00	4.52	3.38E-02	5.91E-02	0.00	0.56	4.55E-01	4.96E-01	0.01	13.22	2.93E-04	1.59E-03*
23	747	T	G	A	A	0.499	0.00	3.29	7.00E-02	1.04E-01	0.01	6.86	8.97E-03	1.29E-02*	0.00	1.54	2.16E-01	2.86E-01	0.01	4.63	3.16E-02	5.35E-02	0.02	16.61	5.00E-05	4.90E-04*
24	760	T	G	S	A*	0.000	0.00	4.06	4.41E-02	6.98E-02	0.03	23.93	1.18E-06	4.46E-06*	0.00	0.71	3.98E-01	4.76E-01	0.00	2.97	8.53E-02	1.13E-01	0.00	2.96	8.55E-02	1.27E-01
25	786	C	T	P	P	0.418	0.03	24.58	8.53E-07	2.61E-06*	0.02	19.38	1.20E-05	3.92E-05*	0.01	10.85	1.03E-03	2.40E-03*	0.01	6.08	1.39E-02	2.83E-02*	0.01	10.39	1.31E-03	4.94E-03*
26	792	G	A	V	V	0.000	0.02	21.85	3.39E-06	9.23E-06*	0.01	5.97	1.47E-02	2.00E-02*	0.02	20.91	5.49E-06	1.58E-05*	0.00	1.12	2.90E-01	3.38E-01	0.00	3.69	5.52E-02	8.72E-02
27	846	G	A/T	T	T	0.000	0.00	2.13	1.45E-01	1.98E-01	0.01	10.09	1.55E-03	2.80E-03*	0.00	1.71	1.92E-01	2.69E-01	0.00	4.25	3.96E-02	6.47E-02	0.00	3.06	8.05E-02	1.23E-01

Table S6. Statistical output for linear models relating pairwise distances of allele frequencies to pairwise difference in environmental proxies (geographical distance, latitude, longitude, altitude and pCPP) in Western Europe. SNP loc.: location of SNP relative to intron 8 – exon 9 boundary. SNP: changed nucleotides. Amino acid: changed amino acids are indicated with an asterisk. FST: F-statistic reflecting overall degree of genetic differentiation. pCPP: predicted critical photoperiod. p.adj: Benjamini-Hochberg adjusted p-value. Significant correlations are marked with an asterisk (p<0.05).

WESTERN EUROPE							geographical distance				latitude				longitude				altitude				pCPP			
#	SNP loc.	SNP		amino acid		F_{ST}	R^2	$F_{1,103}$	*p*	*p-adj*	R^2	$F_{1,103}$	*p*	*p-adj*	R^2	$F_{1,103}$	*p*	*p-adj*	R^2	$F_{1,103}$	*p*	*p-adj*	R^2	$F_{1,15}$	*p*	*p-adj*
-22	-816	A	G			0.695	0.01	0.76	3.84E-01	7.84E-01	0.01	1.21	2.74E-01	6.72E-01	0.11	12.42	6.35E-04	2.59E-03*	0.03	3.60	6.05E-02	3.29E-01	0.01	0.66	4.19E-01	6.41E-01
-21	-764	C	T			0.460	0.02	1.79	1.84E-01	5.62E-01	0.02	2.24	1.37E-01	6.12E-01	0.02	2.34	1.29E-01	2.26E-01	0.00	0.08	7.73E-01	9.47E-01	0.03	3.39	6.84E-02	2.24E-01
-20	-630	C	A/T			0.185	0.06	6.49	1.24E-02	1.01E-01	0.04	4.16	4.39E-02	3.59E-01	0.06	6.28	1.38E-02	4.21E-02*	0.06	7.09	9.00E-03	6.30E-02	0.02	2.17	1.44E-01	3.71E-01
-19	-557	A	C			0.470	0.00	0.07	7.92E-01	9.70E-01	0.00	0.41	5.23E-01	8.54E-01	0.11	13.34	4.10E-04	2.01E-03*	0.00	0.27	6.04E-01	8.46E-01	0.01	0.99	3.23E-01	5.46E-01
-18	-426	T	C			0.887	0.02	2.19	1.42E-01	4.98E-01	0.02	2.05	1.56E-01	6.36E-01	0.02	1.97	1.64E-01	2.51E-01	0.01	1.20	2.76E-01	6.15E-01	0.01	1.33	2.51E-01	5.13E-01
-17	-396	C	A			0.419	0.10	11.11	1.19E-03	1.95E-02*	0.06	6.86	1.01E-02	1.65E-01	0.15	18.04	4.75E-05	3.33E-04*	0.10	10.92	1.31E-03	1.28E-02*	0.02	2.07	1.53E-01	3.76E-01
-16	-392	C	A			0.082	0.02	1.60	2.09E-01	5.69E-01	0.01	0.79	3.77E-01	7.11E-01	0.16	19.80	2.18E-05	1.78E-04*	0.00	0.04	8.36E-01	9.53E-01	0.11	12.25	6.88E-04	3.75E-03*
-15	-385	T	A			0.076	0.00	0.13	7.16E-01	9.74E-01	0.00	0.00	9.82E-01	1.12E+00	0.16	20.32	1.74E-05	2.13E-04*	0.00	0.05	8.26E-01	9.64E-01	0.06	6.11	1.51E-02	5.67E-02
-14	-358	G	T			0.127	0.00	0.08	7.76E-01	9.75E-01	0.00	0.04	8.50E-01	1.10E+00	0.03	3.34	7.07E-02	1.51E-01	0.01	0.73	3.93E-01	6.42E-01	0.00	0.00	9.82E-01	1.00E+00
-13	-312	A	G			0.182	0.02	1.71	1.95E-01	5.61E-01	0.01	0.72	3.98E-01	7.23E-01	0.27	38.40	1.20E-08	5.89E-07*	0.00	0.23	6.32E-01	8.15E-01	0.06	6.10	1.52E-02	5.32E-02
-12	-310	A	G			0.274	0.03	2.85	9.46E-02	4.63E-01	0.01	1.56	2.14E-01	6.17E-01	0.12	14.33	2.58E-04	1.40E-03*	0.04	4.67	3.31E-02	2.03E-01	0.07	7.99	5.65E-03	2.31E-02*
-11	-216	G	A			0.000	0.00	0.02	8.79E-01	1.00E+00	0.00	0.22	6.38E-01	9.20E-01	0.25	24.76	3.94E-06	6.43E-05*	0.02	1.52	2.22E-01	5.18E-01	0.02	1.44	2.34E-01	4.98E-01
-10	-191	T	C			0.000	0.02	1.81	1.83E-01	5.97E-01	0.02	1.87	1.75E-01	6.13E-01	0.03	2.08	1.53E-01	2.50E-01	0.03	2.20	1.42E-01	4.35E-01	0.00	0.03	8.54E-01	1.00E+00
-9	-175	A	T			0.505	0.07	5.53	2.12E-02	1.49E-01	0.05	3.85	5.33E-02	3.73E-01	0.07	5.80	1.84E-02	5.01E-02	0.18	16.15	1.37E-04	2.24E-03*	0.00	0.00	9.82E-01	1.00E+00
-8	-158	G	C			0.171	0.06	7.07	9.09E-03	8.90E-02	0.06	6.04	1.57E-02	1.92E-01	0.14	16.91	7.89E-05	4.83E-04*	0.03	2.68	1.04E-01	5.10E-01	0.45	84.36	4.79E-15	1.17E-13*
-7	-144	G	C			0.310	0.00	0.37	5.46E-01	8.36E-01	0.00	0.43	5.15E-01	8.70E-01	0.02	2.25	1.37E-01	2.31E-01	0.01	1.17	2.82E-01	6.01E-01	0.14	17.05	7.43E-05	6.07E-04*
-6	-143	T	C			0.155	0.03	3.24	7.50E-02	4.59E-01	0.02	2.03	1.57E-01	5.91E-01	0.23	30.52	2.50E-07	6.12E-06*	0.01	0.69	4.07E-01	6.43E-01	0.03	3.18	7.74E-02	2.37E-01
-5	-139	G	C			0.429	0.01	1.19	2.77E-01	6.80E-01	0.00	0.48	4.90E-01	8.57E-01	0.04	4.43	3.78E-02	9.25E-02	0.28	39.87	6.97E-09	3.42E-07*	0.00	0.18	6.71E-01	9.39E-01
-4	-128	T	C			0.151	0.00	0.00	9.44E-01	1.00E+00	0.00	0.00	9.62E-01	1.12E+00	0.02	2.42	1.23E-01	2.24E-01	0.03	2.66	1.06E-01	4.72E-01	0.28	40.56	5.40E-09	8.82E-08*
-3	-98	C	T			0.217	0.02	2.59	1.11E-01	4.52E-01	0.03	2.69	1.04E-01	5.11E-01	0.01	1.56	2.15E-01	3.19E-01	0.02	1.89	1.73E-01	4.24E-01	0.01	1.25	2.67E-01	5.23E-01
-2	-46	A	C			0.000	0.03	2.80	9.74E-02	4.34E-01	0.03	2.98	8.74E-02	4.76E-01	0.04	3.92	5.03E-02	1.12E-01	0.02	2.45	1.21E-01	4.56E-01	0.01	1.13	2.90E-01	5.08E-01
-1	-22	G	A			0.000	0.03	2.89	9.22E-02	5.02E-01	0.03	3.03	8.46E-02	5.18E-01	0.03	2.79	9.81E-02	1.92E-01	0.02	2.34	1.29E-01	4.52E-01	0.01	1.20	2.75E-01	4.99E-01
1	24	C	T	N	N	NA	NA	NA	NA	NA	NA	NA	NA	NA	NA	NA	NA	NA	NA	NA	NA	NA	0.00	NA	NA	NA
2	54	A	G	R	R	NA	NA	NA	NA	NA	NA	NA	NA	NA	NA	NA	NA	NA	NA	NA	NA	NA	0.00	NA	NA	NA
3	66	T	C	N	N	0.112	0.02	2.37	1.27E-01	4.78E-01	0.02	1.68	1.98E-01	6.07E-01	0.00	0.02	9.02E-01	1.00E+00	0.00	0.32	5.73E-01	8.26E-01	0.09	9.90	2.17E-03	9.64E-03*
4	90	G	A	Q	Q	0.000	0.00	0.17	6.82E-01	9.83E-01	0.00	0.39	5.32E-01	8.40E-01	0.02	2.06	1.54E-01	2.43E-01	0.00	0.33	5.65E-01	8.39E-01	0.01	1.24	2.68E-01	5.05E-01
5	111	T	C	S	S	0.249	0.00	0.42	5.20E-01	8.22E-01	0.00	0.21	6.47E-01	9.06E-01	0.00	0.30	5.85E-01	7.17E-01	0.00	0.25	6.20E-01	8.21E-01	0.12	14.39	2.51E-04	1.54E-03*
6	117	C	G/T	G	G	0.000	0.01	0.65	4.22E-01	8.27E-01	0.01	1.17	2.82E-01	6.59E-01	0.07	7.64	6.76E-03	2.37E-02*	0.01	0.79	3.75E-01	6.56E-01	0.00	0.05	8.23E-01	1.00E+00
7	126	A	G	G	G	0.078	0.19	24.39	3.05E-06	1.50E-04*	0.18	23.37	4.69E-06	2.30E-04*	0.06	6.72	1.10E-02	3.58E-02*	0.02	2.25	1.37E-01	4.48E-01	0.59	148.10	2.20E-16	1.08E-14*
8	145	T	C	F	L*	0.000	0.01	0.96	3.30E-01	7.02E-01	0.01	1.31	2.55E-01	6.93E-01	0.03	3.25	7.45E-02	1.52E-01	0.01	0.80	3.75E-01	6.34E-01	0.02	1.71	1.95E-01	4.54E-01
9	159	G	A	S	S	0.216	0.00	0.45	5.04E-01	8.24E-01	0.00	0.24	6.25E-01	9.28E-01	0.00	0.30	5.83E-01	7.32E-01	0.00	0.25	6.15E-01	8.37E-01	0.12	14.58	2.30E-04	1.61E-03*
10	177	C	T	Y	Y	0.000	0.01	0.65	4.23E-01	7.97E-01	0.01	1.16	2.84E-01	6.33E-01	0.07	7.90	5.93E-03	2.24E-02*	0.01	0.85	3.58E-01	6.50E-01	0.00	0.02	8.86E-01	1.00E+00
11	213	T	C	N	N	0.214	0.08	8.61	4.11E-03	5.04E-02	0.05	4.92	2.88E-02	2.82E-01	0.16	20.06	1.95E-05	1.91E-04*	0.08	8.78	3.79E-03	3.10E-02*	0.02	2.33	1.30E-01	3.53E-01
12	249	G	T	T	T	0.178	0.00	0.48	4.92E-01	8.30E-01	0.00	0.27	6.06E-01	9.28E-01	0.01	0.58	4.47E-01	5.76E-01	0.08	8.78	5.53E-01	8.47E-01	0.10	12.01	7.75E-04	3.80E-03*
13	256	G	A	A	T*	NA	NA	NA	NA	NA	NA	NA	NA	NA	NA	NA	NA	NA	NA	NA	NA	NA	0.00	NA	NA	NA
14	291	G	A/C	D	N/H*	0.000	0.00	0.12	7.28E-01	9.65E-01	0.00	0.02	8.96E-01	1.13E+00	0.02	2.47	1.19E-01	2.24E-01	0.00	2.62	1.09E-01	4.45E-01	0.00	0.08	7.79E-01	1.00E+00
15	348	C	T	D	D	NA	NA	NA	NA	NA	NA	NA	NA	NA	NA	NA	NA	NA	NA	NA	NA	NA	0.00	NA	NA	NA
16	375	C	A	I	I	0.123	0.00	0.11	7.40E-01	9.54E-01	0.00	0.17	6.77E-01	9.22E-01	0.01	0.89	3.47E-01	5.00E-01	0.00	NA	NA	NA	0.15	18.03	4.77E-05	4.67E-04*
17	517	C	T/G/A	L	L	0.032	0.01	1.11	2.94E-01	6.56E-01	0.01	0.96	3.30E-01	7.03E-01	0.01	0.71	4.00E-01	5.30E-01	0.01	0.99	1.67E-01	4.31E-01	0.26	31.00	2.70E-07	3.30E-06*
18	555	G	A	S	S	NA	NA	NA	NA	NA	NA	NA	NA	NA	NA	NA	NA	NA	NA	NA	NA	NA	0.00	NA	NA	NA
19	570	C	T	H	H	0.232	0.12	13.88	3.19E-04	7.81E-03*	0.09	10.16	1.90E-03	4.66E-02*	0.11	13.00	4.82E-04	2.15E-03*	0.27	37.98	1.40E-08	3.43E-07*	0.02	2.35	1.28E-01	3.70E-01
20	621	C	T	T	T	0.000	0.00	0.02	8.75E-01	1.00E+00	0.00	0.07	7.88E-01	1.04E+00	0.00	0.24	6.27E-01	7.49E-01	0.13	15.59	1.44E-04	1.76E-03*	0.01	0.97	3.26E-01	5.33E-01
21	729	C	T	H	H	0.000	0.01	1.34	2.50E-01	6.45E-01	0.01	1.24	2.68E-01	6.90E-01	0.00	0.00	9.57E-01	1.00E+00	0.02	2.11	1.49E-01	4.29E-01	0.01	0.86	3.56E-01	5.62E-01
22	731	C	T	A	V*	0.787	0.00	0.50	4.82E-01	8.75E-01	0.01	0.90	3.46E-01	7.05E-01	0.01	0.77	3.83E-01	5.36E-01	0.01	1.12	2.92E-01	5.96E-01	0.00	0.51	4.77E-01	7.08E-01
23	747	T	G	A	A	0.308	0.00	0.50	4.82E-01	8.44E-01	0.01	0.90	3.46E-01	6.77E-01	0.01	0.77	3.83E-01	5.21E-01	0.01	1.12	2.92E-01	5.72E-01	0.00	0.51	4.77E-01	6.87E-01
24	760	T	G	S	A*	0.099	0.01	1.18	2.79E-01	6.52E-01	0.02	1.69	1.97E-01	6.42E-01	0.05	5.62	1.97E-02	5.07E-02	0.02	2.08	1.53E-01	4.17E-01	0.00	0.03	8.56E-01	1.00E+00
25	786	C	T	P	P	NA	NA	NA	NA	NA	NA	NA	NA	NA	NA	NA	NA	NA	0.00	NA	NA	NA	0.00	NA	NA	NA
26	792	G	A	V	V	0.168	0.00	0.14	7.12E-01	9.97E-01	0.00	0.01	9.43E-01	1.00E+00	0.06	6.07	1.54E-02	4.44E-02	0.00	0.16	6.94E-01	8.72E-01	0.00	0.13	7.23E-01	9.84E-01
27	846	G	A/T	T	T	0.035	0.00	0.20	6.54E-01	9.71E-01	0.00	0.01	9.14E-01	1.00E+00	0.04	4.08	4.60E-02	1.07E-01	0.00	0.05	8.23E-01	9.84E-01	0.01	1.51	2.21E-01	4.93E-01

Table S7. Statistical output for linear models relating pairwise distances of allele frequencies to pairwise difference in environmental proxies (geographical distance, latitude, longitude, altitude and pCPP) in Eastern Europe. SNP loc.: location of SNP relative to intron 8 – exon 9 boundary. SNP: changed nucleotides. Amino acid: changed amino acids are indicated with an asterisk. F_{ST}: F-statistic reflecting overall degree of genetic differentiation. pCPP: predicted critical photoperiod. *p*.adj: Benjamini-Hochberg adjusted p-value. Significant correlations are marked with an asterisk ($p<0.05$).

EASTERN EUROPE							geographical distance				latitude				longitude				altitude				pCPP			
#	SNP loc.	SNP		amino acid		F_{ST}	R^2	$F_{1,375}$	*p*	*p-adj*	R^2	$F_{1,375}$	*p*	*p-adj*	R^2	$F_{1,375}$	*p*	*p-adj*	R^2	$F_{1,375}$	*p*	*p-adj*	R^2	$F_{1,375}$	*p*	*p-adj*
-22	-816	A	G			NA	NA	NA	NA	NA	NA	NA	NA	NA	NA	NA	NA	NA	NA	NA	NA	NA	0.00	NA	NA	NA
-21	-764	C	T			0.241	0.04	10.29	1.53E-03	2.50E-02*	0.06	14.82	1.53E-04	1.50E-03*	0.04	8.43	4.05E-03	3.31E-02	0.02	5.77	1.71E-02	1.67E-01	0.04	9.25	2.63E-03	1.61E-04*
-20	-630	C	A/T			NA	NA	NA	NA	NA	NA	NA	NA	NA	NA	NA	NA	NA	NA	NA	NA	NA	0.00	NA	NA	NA
-19	-557	A	C			NA	NA	NA	NA	NA	NA	NA	NA	NA	NA	NA	NA	NA	NA	NA	NA	NA	0.00	NA	NA	NA
-18	-426	T	C			NA	NA	NA	NA	NA	NA	NA	NA	NA	NA	NA	NA	NA	NA	NA	NA	NA	0.00	NA	NA	NA
-17	-396	C	A			NA	NA	NA	NA	NA	NA	NA	NA	NA	NA	NA	NA	NA	NA	NA	NA	NA	0.00	NA	NA	NA
-16	-392	C	A			0.119	0.00	0.12	7.27E-01	1.00E+00	0.01	2.91	8.90E-02	2.42E-01	0.00	0.32	5.73E-01	9.06E-01	0.00	1.39	2.39E-01	4.68E-01	0.01	2.20	1.39E-01	3.69E-02*
-15	-385	T	A			0.682	0.01	2.44	1.19E-01	3.44E-01	0.09	39.04	1.13E-09	2.77E-08*	0.02	6.04	1.44E-02	6.43E-02	0.01	4.57	3.31E-02	1.47E-01	0.00	1.81	1.79E-01	5.84E-02
-14	-358	G	T			0.769	0.04	15.76	8.64E-05	2.12E-03*	0.04	13.73	2.43E-04	1.99E-03*	0.02	7.27	7.32E-03	4.45E-02	0.02	7.21	7.57E-03	9.27E-02	0.05	17.70	3.25E-05	1.32E-06*
-13	-312	A	G			0.160	0.01	1.95	1.63E-01	4.21E-01	0.08	33.09	1.83E-08	2.98E-07*	0.01	5.46	2.00E-02	6.54E-02	0.01	3.70	5.52E-02	1.93E-01	0.00	1.83	1.77E-01	5.06E-02
-12	-310	A	G			0.673	0.00	0.06	8.14E-01	1.00E+00	0.00	0.19	6.61E-01	1.00E+00	0.00	0.46	4.99E-01	8.73E-01	0.00	0.11	7.38E-01	1.00E+00	0.00	0.56	4.55E-01	2.32E-01
-11	-216	G	A			0.206	0.00	1.02	3.14E-01	6.40E-01	0.00	1.84	1.76E-01	4.31E-01	0.00	0.31	5.76E-01	8.82E-01	0.00	0.00	9.74E-01	1.00E+00	0.01	3.14	7.71E-02	1.26E-02*
-10	-191	T	C			0.691	0.01	2.25	1.35E-01	3.67E-01	0.00	0.29	5.89E-01	1.00E+00	0.02	7.29	7.27E-03	4.45E-02	0.01	5.54	1.91E-02	1.56E-01	0.01	3.93	4.82E-02	4.92E-03*
-9	-175	A	T			0.211	0.01	3.99	4.66E-02	1.76E-01	0.00	0.97	3.24E-01	7.56E-01	0.02	5.96	1.51E-02	5.69E-02	0.01	5.33	2.15E-02	1.31E-01	0.00	1.73	1.90E-01	6.96E-02
-8	-158	G	C			0.767	0.01	3.74	5.37E-02	1.65E-01	0.10	43.16	1.69E-10	8.27E-09*	0.01	4.39	3.68E-02	1.00E-01	0.12	49.37	1.00E-11	4.91E-10*	0.01	2.88	9.04E-02	1.66E-02*
-7	-144	G	C			0.166	0.02	8.27	4.26E-03	3.48E-02*	0.02	6.97	8.66E-03	3.86E-02*	0.01	5.45	2.01E-02	5.81E-02	0.07	26.95	3.43E-07	8.41E-06*	0.01	4.32	3.85E-02	4.92E-03*
-6	-143	T	C			0.197	0.00	0.15	6.96E-01	1.00E+00	0.02	7.60	6.14E-03	3.01E-02*	0.01	2.30	1.30E-01	2.90E-01	0.01	3.09	7.94E-02	1.95E-01	0.00	0.22	6.37E-01	3.64E-01
-5	-139	G	C			NA	NA	NA	NA	NA	NA	NA	NA	NA	NA	NA	NA	NA	NA	NA	NA	NA	0.00	NA	NA	NA
-4	-128	T	C			0.235	0.00	0.03	8.62E-01	1.00E+00	0.01	5.33	2.16E-02	8.12E-02	0.01	2.54	1.12E-01	2.61E-01	0.01	4.44	3.58E-02	1.46E-01	0.00	1.57	2.11E-01	8.19E-02
-3	-98	C	T			NA	NA	NA	NA	NA	NA	NA	NA	NA	NA	NA	NA	NA	NA	NA	NA	NA	0.00	NA	NA	NA
-2	-46	A	C			0.033	0.01	3.78	5.26E-02	1.72E-01	0.01	3.19	7.48E-02	2.16E-01	0.01	2.17	1.41E-01	2.77E-01	0.01	4.94	2.68E-02	1.31E-01	0.01	2.40	1.22E-01	2.75E-02*
-1	-22	G	A			NA	NA	NA	NA	NA	NA	NA	NA	NA	NA	NA	NA	NA	NA	NA	NA	NA	0.00	NA	NA	NA
1	24	C	T	N	N	0.022	0.00	0.05	8.17E-01	1.00E+00	0.00	0.78	3.78E-01	8.43E-01	0.00	0.05	8.21E-01	1.00E+00	0.01	3.43	6.47E-02	1.98E-01	0.00	0.00	9.74E-01	6.56E-01
2	54	A	G	R	R	0.046	0.00	0.05	8.17E-01	1.00E+00	0.00	0.78	3.78E-01	8.06E-01	0.00	0.05	8.21E-01	1.00E+00	0.01	3.43	6.47E-02	1.86E-01	0.00	0.00	9.74E-01	6.76E-01
3	66	T	C	N	N	0.169	0.01	5.22	2.29E-02	1.40E-01	0.00	0.28	6.00E-01	1.00E+00	0.02	9.32	2.42E-03	2.97E-02	0.01	5.19	2.33E-02	1.27E-01	0.01	2.87	9.12E-02	1.86E-02*
4	90	G	A	Q	Q	NA	NA	NA	NA	NA	NA	NA	NA	NA	NA	NA	NA	NA	NA	NA	NA	NA	0.00	NA	NA	NA
5	111	T	C	S	S	0.216	0.00	1.59	2.08E-01	4.86E-01	0.00	0.13	7.19E-01	1.00E+00	0.01	3.76	5.31E-02	1.30E-01	0.00	1.47	2.26E-01	4.82E-01	0.01	2.22	1.37E-01	3.35E-02*
6	117	C	G/T	G	G	0.047	0.00	0.11	7.38E-01	1.00E+00	0.00	0.09	7.65E-01	1.00E+00	0.00	0.32	5.71E-01	9.32E-01	0.00	0.13	7.23E-01	1.00E+00	0.00	1.79	1.82E-01	6.32E-02
7	126	A	G	G	G	0.751	0.00	0.00	9.88E-01	1.00E+00	0.03	10.91	1.05E-03	6.43E-03*	0.02	6.03	1.45E-02	5.91E-02	0.00	0.80	3.73E-01	6.52E-01	0.00	0.05	8.26E-01	5.22E-01
8	145	T	C	F	L*	0.712	0.02	7.55	6.28E-03	4.39E-02*	0.01	2.06	1.52E-01	3.92E-01	0.02	7.73	5.72E-03	4.00E-02	0.01	2.90	8.97E-02	2.09E-01	0.00	0.13	7.17E-01	4.24E-01
9	159	G	A	S	S	0.535	0.01	4.46	3.53E-02	1.57E-01	0.00	0.09	7.61E-01	1.00E+00	0.02	8.70	3.39E-03	3.32E-02	0.00	0.00	9.83E-01	1.00E+00	0.00	0.49	4.84E-01	2.57E-01
10	177	C	T	Y	Y	0.186	0.00	1.25	2.64E-01	5.87E-01	0.00	0.27	6.02E-01	1.00E+00	0.00	0.86	3.54E-01	6.67E-01	0.00	0.55	4.59E-01	7.76E-01	0.00	0.04	8.50E-01	5.55E-01
11	213	T	C	N	N	NA	NA	NA	NA	NA	NA	NA	NA	NA	NA	NA	NA	NA	NA	NA	NA	NA	0.00	NA	NA	NA
12	249	G	T	T	T	0.211	0.00	0.73	3.93E-01	7.71E-01	0.00	0.35	5.55E-01	1.00E+00	0.01	4.32	3.83E-02	9.87E-02	0.00	1.28	2.58E-01	4.87E-01	0.00	1.24	2.66E-01	1.25E-01
13	256	G	A	A	T*	0.951	0.01	3.82	5.13E-02	1.80E-01	0.00	0.77	3.81E-01	7.78E-01	0.02	6.07	1.42E-02	6.97E-02	0.00	1.75	1.87E-01	4.17E-01	0.00	0.65	4.19E-01	2.05E-01
14	291	G	A/C	D	N/H*	NA	NA	NA	NA	NA	NA	NA	NA	NA	NA	NA	NA	NA	NA	NA	NA	NA	0.00	NA	NA	NA
15	348	C	T	D	D	0.213	0.00	0.61	4.36E-01	8.22E-01	0.01	4.18	4.15E-02	1.36E-01	0.00	0.36	5.52E-01	9.32E-01	0.02	7.46	6.59E-03	1.08E-01	0.00	1.25	2.64E-01	1.19E-01
16	375	C	A	I	I	0.265	0.01	5.06	2.51E-02	1.23E-01	0.00	0.22	6.40E-01	1.00E+00	0.02	9.46	2.26E-03	3.69E-02	0.00	0.13	7.24E-01	1.00E+00	0.00	1.41	2.36E-01	9.61E-02
17	517	C	T/G/A	L	L	NA	NA	NA	NA	NA	NA	NA	NA	NA	NA	NA	NA	NA	NA	NA	NA	NA	0.00	NA	NA	NA
18	555	G	A	S	S	0.193	0.02	8.48	3.82E-03	3.74E-02*	0.01	3.61	5.84E-02	1.79E-01	0.01	5.60	1.85E-02	6.46E-02	0.00	1.43	2.32E-01	4.74E-01	0.00	0.42	5.16E-01	2.84E-01
19	570	C	T	H	H	NA	NA	NA	NA	NA	NA	NA	NA	NA	NA	NA	NA	NA	NA	NA	NA	NA	0.00	NA	NA	NA
20	621	C	T	T	T	0.092	0.01	1.95	1.63E-01	4.00E-01	0.08	33.09	1.83E-08	2.24E-07*	0.01	5.46	2.00E-02	6.13E-02	0.01	3.70	5.52E-02	1.80E-01	0.00	1.83	1.77E-01	5.42E-02
21	729	C	T	H	H	0.150	0.11	46.26	4.10E-11	2.01E-09*	0.02	6.96	8.71E-03	3.55E-02*	0.14	60.95	5.92E-14	2.90E-12	0.01	3.99	4.66E-02	1.75E-01	0.11	44.46	9.33E-11	1.90E-12*
22	731	*C	T	A	V*	NA	NA	NA	NA	NA	NA	NA	NA	NA	NA	NA	NA	NA	NA	NA	NA	NA	0.00	NA	NA	NA
23	747	T	G	A	A	0.676	0.00	1.08	3.00E-01	6.40E-01	0.00	0.03	8.58E-01	1.00E+00	0.01	2.29	1.31E-01	2.78E-01	0.00	1.07	3.01E-01	5.47E-01	0.00	0.07	7.92E-01	4.85E-01
24	760	T	G	S	A*	0.136	0.01	4.24	4.02E-02	1.64E-01	0.02	8.24	4.34E-03	2.36E-02*	0.00	0.56	4.53E-01	8.22E-01	0.01	5.54	1.92E-02	1.34E-01	0.01	3.61	5.84E-02	7.15E-03*
25	786	C	T	P	P	0.058	0.02	8.57	3.63E-03	4.44E-02*	0.03	11.52	7.61E-04	5.32E-03*	0.01	2.26	1.33E-01	2.72E-01	0.01	3.27	7.12E-02	1.84E-01	0.01	3.38	6.68E-02	9.54E-03*
26	792	G	A	V	V	0.126	0.01	5.19	2.33E-02	1.27E-01	0.01	4.40	3.65E-02	1.28E-01	0.04	13.65	2.53E-04	6.19E-03	0.01	3.35	6.80E-02	1.85E-01	0.00	1.35	2.46E-01	1.05E-01
27	846	G	A/T	T	T	NA	NA	NA	NA	NA	NA	NA	NA	NA	NA	NA	NA	NA	NA	NA	NA	NA	0.00	NA	NA	NA
